# Genetic Signatures in the Envelope Glycoproteins of HIV-1 that Associate with Broadly Neutralizing Antibodies

**DOI:** 10.1371/journal.pcbi.1000955

**Published:** 2010-10-07

**Authors:** S. Gnanakaran, Marcus G. Daniels, Tanmoy Bhattacharya, Alan S. Lapedes, Anurag Sethi, Ming Li, Haili Tang, Kelli Greene, Hongmei Gao, Barton F. Haynes, Myron S. Cohen, George M. Shaw, Michael S. Seaman, Amit Kumar, Feng Gao, David C. Montefiori, Bette Korber

**Affiliations:** 1 Theoretical Biology and Biophysics, Los Alamos National Laboratory, Los Alamos, New Mexico, United States of America; 2 Santa Fe Institute, Santa Fe, New Mexico, United States of America; 3 Center for Nonlinear Studies, Los Alamos National Laboratory, Los Alamos, New Mexico, United States of America; 4 Department of Surgery, Duke University Medical Center, Durham, North Carolina, United States of America; 5 Department of Medicine, Duke University Medical Center, Durham, North Carolina, United States of America; 6 Department of Medicine, University of North Carolina at Chapel Hill, Chapel Hill, North Carolina, United States of America; 7 Department of Medicine, University of Alabama at Birmingham, Birmingham, Alabama, United States of America; 8 Department of Microbiology, University of Alabama at Birmingham, Birmingham, Alabama, United States of America; 9 Division of Viral Pathogenesis, Beth Israel Deaconess Medical Center, Boston, Massachusetts, United States of America; Imperial College London, United Kingdom

## Abstract

A steady increase in knowledge of the molecular and antigenic structure of the gp120 and gp41 HIV-1 envelope glycoproteins (Env) is yielding important new insights for vaccine design, but it has been difficult to translate this information to an immunogen that elicits broadly neutralizing antibodies. To help bridge this gap, we used phylogenetically corrected statistical methods to identify amino acid signature patterns in Envs derived from people who have made potently neutralizing antibodies, with the hypothesis that these Envs may share common features that would be useful for incorporation in a vaccine immunogen. Before attempting this, essentially as a control, we explored the utility of our computational methods for defining signatures of complex neutralization phenotypes by analyzing Env sequences from 251 clonal viruses that were differentially sensitive to neutralization by the well-characterized gp120-specific monoclonal antibody, b12. We identified ten b12-neutralization signatures, including seven either in the b12-binding surface of gp120 or in the V2 region of gp120 that have been previously shown to impact b12 sensitivity. A simple algorithm based on the b12 signature pattern was predictive of b12 sensitivity/resistance in an additional blinded panel of 57 viruses. Upon obtaining these reassuring outcomes, we went on to apply these same computational methods to define signature patterns in Env from HIV-1 infected individuals who had potent, broadly neutralizing responses. We analyzed a checkerboard-style neutralization dataset with sera from 69 HIV-1-infected individuals tested against a panel of 25 different Envs. Distinct clusters of sera with high and low neutralization potencies were identified. Six signature positions in Env sequences obtained from the 69 samples were found to be strongly associated with either the high or low potency responses. Five sites were in the CD4-induced coreceptor binding site of gp120, suggesting an important role for this region in the elicitation of broadly neutralizing antibody responses against HIV-1.

## Author Summary

Neutralizing antibodies block infection of cells and thus are considered important to elicit with vaccines. A central problem in HIV-1 vaccine design is that HIV-1 is extremely variable and employs a number of strategies to avoid being recognized by antibodies. Despite this, a subset of infected individuals mounts potent, cross-reactive neutralizing antibody responses. We developed computational strategies for identifying correlations between mutational patterns in the HIV-1 envelope glycoproteins (gp120 and gp41) and neutralization phenotypes. We first applied these methods to define mutations that correlated with susceptibility to the potent neutralizing antibody b12, as a means to explore the appropriateness of applying our computational strategies to neutralizing antibody phenotypes within the well-understood context of b12-gp120 interactions. Signature sites of known importance were found. We then defined signatures in a panel of envelope glycoproteins sampled from HIV-1-infected individuals who made either potent or weak neutralizing antibody responses, with the hypothesis that common features of the envelope glycoproteins that elicit good antibodies in natural infection might be useful to incorporate as vaccine immunogens. Signature mutations associated with potent neutralizing antibody responses were concentrated in the coreceptor binding site of gp120 – a key region for HIV-1 entry into cells.

## Introduction

Elicitation of broadly cross-reactive neutralizing antibody (NAb) responses is a high priority for HIV-1 vaccines [1]–[4]. Many candidate immunogens elicit strong NAb responses against highly neutralization-sensitive strains of HIV-1; however, these vaccine-elicited antibodies neutralize very few circulating strains [5]–[7] and have not afforded protection in past human efficacy trials [8]–[10]. A recently completed efficacy trial in Thailand (RV144), in which a modest reduction in the rate of HIV-1 infection was observed [11], provides hope that with further improvements a more acceptable level of efficacy is obtainable. It is too soon to know whether NAbs contributed to the observed efficacy in RV144. Based on immunogenicity data from earlier phase I and II clinical trials of this and related vaccines [4], [12], improved NAb responses may be one way to achieve greater protection. Such improvements are likely to require novel vaccinedesigns.

Most current efforts to design NAb-based HIV-1 vaccine immunogens are guided in part by knowledge of the molecular structure of the viral Envelope (Env) glycoproteins that serve as the sole targets for NAbs [13]–[16]. These Env glycoproteins consist of a surface gp120 and transmembrane gp41 that associate non-covalently and assemble into a trimeric complex of gp120-gp41 heterodimers on the virus surface, where the mature Env trimer spike mediates virus entry into host cells [17]–[19]. Entry is mediated by successive binding of gp120 to its cellular CD4 receptor and an obligatory coreceptor, most often the chemokine receptor CCR5, triggering conformational changes that permit gp41 to induce membrane fusion [18]–[20]. Env trimers and their individual constituents are genetically variable, conformationally flexible and heavily glycosylated, making them difficult targets for NAbs [1], [2], [19], [21]. Because fitness constraints do not permit the virus to evolve to become completely resistant to neutralization [22], [23], certain NAb epitopes remain vulnerable that are of particular interest for vaccine development. Some of these epitopes are well studied, whereas others remain unknown or only partially characterized [2], [4], [24].

The structural complexity of Env requires sophisticated methods for the analysis of NAb epitopes. X-ray crystallography and cryo-electron tomography, together with data from mutagenesis and biophysical studies, have been used to illuminate several vulnerable regions in great detail. Examples of how this information is used for novel immunogen designs include the optimization and stabilization of epitopes in the receptor and coreceptor binding regions of gp120 [25]–[27]. Other examples include innovative structural variants of gp41 [28]–[30] and optimal mimics of gp120 and gp41 epitopes recognized by broadly neutralizing monoclonal antibodies (mAbs) [31]–[35]. Although these new design efforts are in early stages of testing, none so far have yielded substantial improvements.

Many new concepts for NAb-inducing vaccines based on HIV-1 Env are being explored. These concepts are complicated by inconsistencies between the antigenic and immunogenic properties of key epitopes. For example, Env antigens that possess high affinity epitopes for broadly neutralizing mAbs fail to elicit these types of antibodies [28]–[30], [36]–[39]. Also, gp120 antigens similar to those that performed poorly as early vaccine candidates contain epitopes that are capable of absorbing-out a substantial fraction of broadly NAbs in sera from a subset of HIV-1-infected individuals [40]–[43]. Some B cell responses might be down regulated by self-tolerance mechanisms, as has been suggested for epitopes in the membrane proximal external region (MPER) of gp41 [44], [45]. Other B cell responses might be down regulated by immunosuppressive properties of gp120 [46]–[48]. Although it remains unclear why some of the most attractive Env epitopes are poor immunogens, the potent neutralizing activities of a subset of human mAbs [49], [50] and sera from HIV-1-infected individuals [51] suggest it might be possible to design better vaccine immunogens.

A greater understanding of the antigenic and immunogenic properties of Env should facilitate the discovery of an effective HIV-1 vaccine. We (and others) are using computational analyses of large neutralization datasets derived from assays with HIV-1-positive sera and molecularly cloned Env-pseudotyped viruses to gain new insights. Statistically significant associations are sought between amino acids in particular positions in the alignment and either i) the neutralization susceptibility of a given Env, or ii) the potency and cross-reactivity of neutralizing antibody responses of individuals harboring a given Env. Here we use the term “signatures” to refer to the amino acids in a given position in Env that are associated with a neutralization phenotype. Previously, several amino acid signatures in gp120 and gp41 were identified that strongly associate with the antigenic determinants of NAbs in sera from HIV-1-infected subjects [52], [53]. Such signatures could either be a consequence of direct contacts for NAbs, or reflect conformational requirements/constraints that regulate Ab access.

Because of the distinctive lineages in HIV evolution, found at multiple levels (clades, subclades, and geographic clusters within a clade), it is critical to correct for the phylogenetic associations among sequences when defining signatures rather than merely attempting to predict phenotypes. Not accounting for phylogeny can lead to spurious positive signals that result from lineage effects and a reduced sensitivity, as was seen when associations were sought between host HLA and HIV amino acid substitutions at the population level [54], [55]. We used three distinct phylogenetically corrected statistical approaches to look for signature patterns. The first was the approach taken by Bhattacharya et al. [54], but modified to enable looking at combinations of sites and combinations of amino acids within sites. The two novel statistical strategies for defining signatures used here are conditional mutual information and a modified decision forest approach. We first tested our computational signature identification methods in the context of neutralizing antibody signatures by accurately identifying a subset of the known determinants of the epitope for broadly neutralizing mAb b12. Here our primary goal was not prediction of the phenotype of unknown sequences; rather the main goals were to identify amino acid mutational patterns that correlate with b12 sensitivity independently of founder effects, and then form hypotheses regarding sites/mutations that may directly impact neutralization, to use biological knowledge available in the literature to evaluate these hypotheses, and to suggest further experiments to validate sites/mutations for which knowledge is presently lacking. The b12 signature patterns we identified were well supported by the literature, indicating the computational methods were indeed identifying meaningful sites. We then applied these methods in a reciprocal fashion to determine whether amino acid signatures in the Env proteins from HIV-1 infected individuals with particularly broad NAb responses could be identified relative to individuals who do not elicit broad responses. Our hypothesis was that there may be common features in Envs capable of eliciting potent neutralizing antibodies, and identification of such signatures may ultimately be helpful for immunogen design. Our findings suggest that broadly NAb responses are determined in part by features in the CD4-induced (CD4i) co-receptor binding site (CoRbs) of gp120.

## Results

### Identification of signature sites and mutational patterns associated with b12 susceptibility

**Figure 1:**
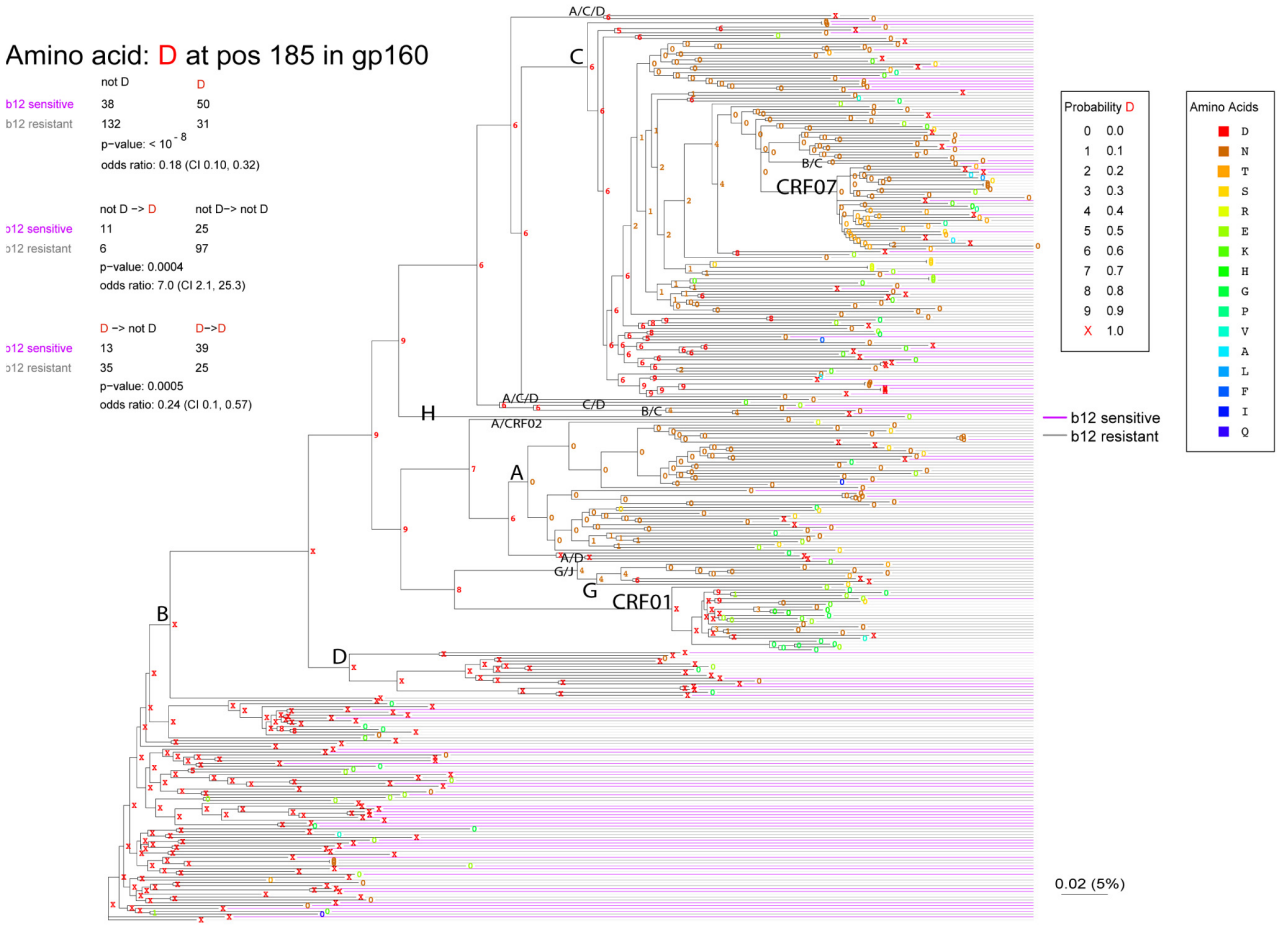
Maximum Likelihood tree of the Env sequences drawn to illustrate phylogenetically corrected signature sites identification in the framework of this data used in the b12 study. The example shown here is the signature amino acid Aspartic acid “D” at position 185. A D at position 185 is shown in red. When a leaf taxa is a “D” in position 185, it is *known* to be a D at the node, and so given the probability of 1, and represented by a red X (see the legend). If the amino acid at position 185 is not a D in a leaf node, it has a probability of 0 of being D, and the 0 is written in the color of the observed amino acid, as shown in the key. The probability of an ancestral node being “D” is indicated throughout the tree, and the color used to write the probability indicates the most probable amino acid at position 185 at that node. 319 Envs are included in the tree, of which 251 were matched to b12 phenotypes for use in defining the signature sites; b12 sensitive Envs are indicated by magenta lines, b12 resistant Envs are indicated by gray. The Envs that were kept blinded during the initial signature defining procedures, and used for later as a test set, are indicated by very light gray lines. An uncorrected contingency table is shown at the top, and an Aspartic acid (D) at position 185 is very strongly associated with b12 susceptibility. Beneath that is shown the phylogenetically corrected signature analysis, that each support this association, indicating it is not merely an artifact of one clade within the tree being more susceptible to b12. This is a real concern: D is very common in the B clade, and the B clade is the clade most susceptible to b12, while D is far less common in other clades, which are all less frequently susceptible to b12, and so the uncorrected association in the top contingency table might be a lineage effect. However, throughout the entire tree, when an Env has mutated towards a D at the tip in this position (not D to D), the Env at the tip tends to be susceptible to b12, but when it moves away from it, (D to not D), the Env tends to be resistant, suggesting a direct association between b12 susceptibility and this site.

Neutralization data and Env sequences relating to the b12 epitope that overlaps the CD4 binding site (CD4bs) of gp120 [35] were analyzed as a means to partially validate our computational methods for signature site identification. The mAb b12 was chosen for methodological validation purposes both because many details regarding its epitope are known, and because it is an epitope of great interest for vaccine design. The analyses utilized genetic sequences and b12 sensitivities of 251 clonal Env-pseudotyped viruses representing many HIV subtypes, recombinant lineages and disease stages (Fig. 1, Table S1). IC50 values were determined from neutralization curves where the highest dose of b12 tested was either 25 µg/ml or 50 µg/ml, depending on the experiment. Viruses not neutralized at the highest dose tested are referred to here as being resistant; that is not to say, however, that some of the viruses would not have been neutralized by higher b12 concentrations. Among the 251 viruses tested, 88 (35%) were sensitive at varying levels (Table S2), and the other 163 were resistant at the highest concentration tested.

**Figure 2:**
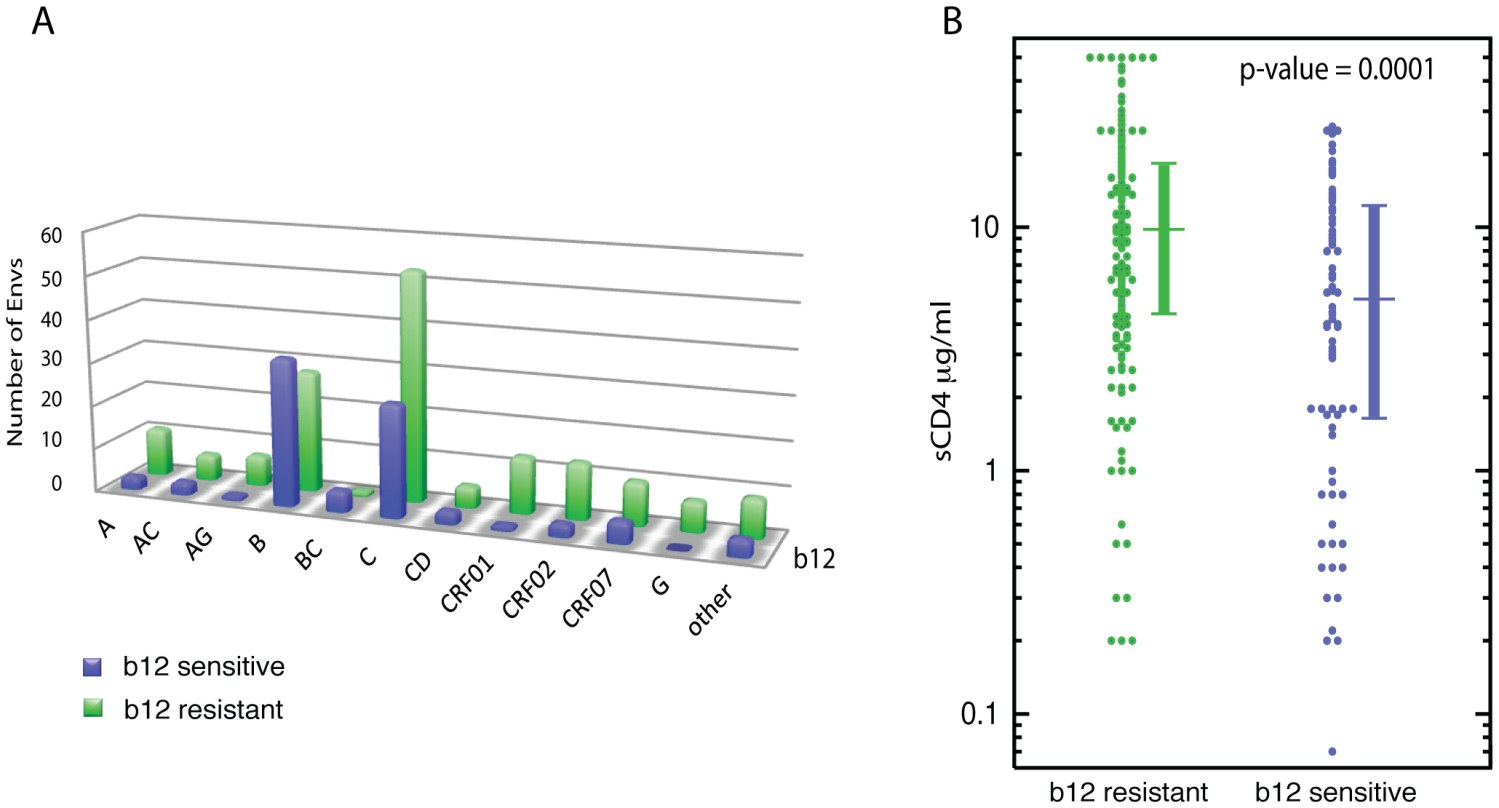
Correlates of b12 sensitivity. (**A**) Counts of b12 sensitive and resistant viruses grouped by subtype, intersubtype recombinant and circulating recombinant forms (CRFs). Sparsely represented subtypes D and G, CRF14, and unique recombinant Envs were grouped into the “other” category. The only 2 subtype categories with greater numbers of b12 sensitive than resistant Envs were subtype B and B/C recombinants (in these two cases the green bars are higher than purple). A Kruskal-Wallace non-parametric comparison of all groups indicated that at least one subtype was distinctive (p = 0.033). A comparison of the B subtype Envs versus all others indicated that they were far more likely to be sensitive to neutralization by b12 (Fisher's exact p-value 3.7×10^−5^, odds ratio 3.6, 95% confidence interval: 1.9, 6.9). (**B**) sCD4 susceptibility is greater among b12 sensitive viruses. The amount of sCD4 required for 50% neutralization was greater among b12 resistant viruses (Wilcoxon rank test, p = 0.0001, median and interquartile range shown to the right of each distribution, median 9.8 µg/ml among b12 resistant viruses, median 5.1 µg/ml among sensitive viruses).

First, other potential correlates of b12 sensitivity were examined, including viral genetic subtype, sensitivity to soluble CD4 (sCD4), and the disease stage of the donor at the time of virus isolation. Multiple subtypes were included in the study (Fig. 1). Envs that were B subtype exhibited the highest frequency of b12 neutralization susceptibility (Fisher's exact test p = 3.6×10^−4^, comparing B subtype to all others, Fig. 2A). In situations like this, in which there is a strong clade structure in the evolutionary tree and an enrichment of the phenotype of interest in a particular clade, it is critical to employ strategies that include phylogenetic correction to avoid spurious positives when seeking amino acid signatures. This is because it is likely that only a small number out of the amino acids that are commonly enriched in the B clade will directly impact an Envelope's susceptibility to b12; however, any amino acid enriched in the B clade, including amino acids that are common in the B clade due to founder effects, will be biased towards appearing associated with b12 sensitivity (Fig. 1).

Envs of the target viruses were obtained and sequenced at different stages of infection. The Fiebig stage [56] for most subjects at the time the Env was sampled was experimentally determined as an indicator of stage of infection (Table S1). When the Fiebig stage was not experimentally determined, the subjects were generally noted to be in a “chronic” or “early/acute” stage at the time the sample was obtained (Table S1). When the subjects were broken into categories of “chronic” (grouping those in Fiebig stages VI or V/VI, with those noted to be in chronic infection) and “early” (grouping Fiebig stages I-V, with those noted to be in acute or early infection) there was no difference between b12 sensitivity or resistance, nor was there any correlation between b12 sensitivity and the series of Fiebig stages (data not shown). Thus the results from our cross-sectional examination of b12 resistance at different stages of infection suggests that the emergence of b12 resistance over time that was previously observed in a longitudinal study in a small number of subjects [57] may not be a common pattern. Finally, consistent with previous findings [58], Envs that were susceptible to b12 neutralization were more sensitive to neutralization by sCD4 (p = 0.0001, Wilcoxon rank sum test, Fig. 2B). Among just the b12 sensitive viruses, there was a weak correlation between the neutralizing potencies of b12 and sCD4 (Kendall tau Rank Correlation: p = 0.0015, tau = 0.23, data not shown).

Our signature analyses strategies identified ten b12 sensitivity amino acid signatures in Env. Associations with a q value (false discovery rate)<0.2 are presented in Tables 1 and 2; a relatively high q value cut off was used to be inclusive at this hypothesis forming stage. Seven signatures (6 in gp120 and 1 in gp41) were identified by phylogenetically corrected contingency table analysis [54] (example shown in Fig. 1). Specific amino acid mutational patterns in each position formed the basis of contingency table analysis; these are noted in Tables 1 and 2. We used several different likelihood trees as input to test the sensitivity of the signature analysis results to the phylogenetic tree. Two distinct tree topologies from two different runs on parallel computers (see Materials and Methods section) gave identical signature results in terms of sites and amino acids, with, as expected, slightly different p and q-values. A tree generated using PHYML [59], a program that can be run very quickly but had a less optimal likelihood scores than our trees based on a more extensive exploration of the tree topologies, identified 6/7 of the original signature sites, but missed the site 651 (which we have since shown experimentally to impact b12 sensitivity, see below), and captured two other sites (site 364 and 742) with very borderline q-values (∼0.19). Given the intrinsic variation in the trees, and our inclusive high q value cutoff of 0.2 for hypothesis generation, one would expect some run-to-run variation. The overall consistency of the signature results based on the 3 trees, however, suggests the results are relatively robust and independent of the tree; we present in Tables 1 and 2 the sites and statistics based on the tree with the maximum likelihood.

**Table 1:** Sites identified as signatures of b12 sensitivity using any of the three signature-defining approaches: contingency table, CMI, and ensemble machine learning method.

HXB2 position	Signature Region	CMI^1^	Fisher's^2^ Sensitive/Resistant	Recurrent top splits in decision trees^3^
**gp120**				
163	V2	Yes	-	-
173	V2	Yes	**Y/HS**	**Y→!Y** (32)
182	V2	Yes	**-**	**-**
185	V2	Yes	**DEN/GST**	**D→!D** (38), **!D→D** (59)
268	outer domain	Yes	**ES/KR**	**E→!E** (83)
364	b12	No	**PS/AH**	**-**
369	b12	No	**AP/ILQ**	**-**
461	b12	No	**EP**	**E→!E** (61)
**gp41**				
651	C-heptad repeat	No	**N/DIS**	**N→!N** (17)
655	C-heptad repeat	Yes	**-**	**-**

1 The CMI approach does not provide specific information regarding which amino acids give rise to the signal, although particularly distinctive substitutions can be seen by examining the data (Sup. Fig. 1). A “Yes” in the CMI column means the site was associated with b12 sensitivity or resistance.

2 The Fisher's exact contingency table is based on specific amino acids or sets of amino acids, such that the amino acids associated with signature sites are explicit; and amino acids associated with b12 resistance are underlined, whereas amino acids associated with b12 susceptibility are *not* underlined.

3 The recurrent top splits in the decision trees (the number in parentheses indicates how many times it was found) provide information about the key signature amino acid substitutions. The exclamation point (!) means “not” in these tables and figures, thus E→!E means that “E” is found in the immediate ancestral state of the sequence, and is “not E” in the sequence.

**Table 2:** Summary of statistics of signature sites of b12 sensitivity.

HXB2 position^1^	Amino acid^2^	Statistic^3^	p-value^4^	q-value^4^	Odds ratio^4^	r1c1 Sensitive Change^5^	r1c2 Sensitive Stable^5^	r2c1 Resistant Change^5^	r2c2 Resistant Stable^5^	Strength^6^	Test^7^
163		CMI	<10^−3^	<10^−3^							1aa
173	*Y*→!Y	Fisher	0.00024	0.042	0.14	6	74	40	105	0.2413	1aa
173	!HS→HS	Fisher	0.0017	0.0087	0.18	3	78	27	123	0.2242	>1aa
173		CMI	0.001	0.013							1aa
182		CMI	0.002	0.12							1aa
**185** 8	**!D→** ***D***	**Fisher**	**0.00036**	**0.04**	**6.98**	**11**	**25**	**6**	**97**	**6.4615**	**1aa**
**185**	***D*** **→!D**	**Fisher**	**0.00053**	**0.059**	**0.24**	**13**	**39**	**35**	**25**	**0.2528**	**1aa**
185	*DEN*→!DEN	Fisher	4.4×10^−7^	0.00013	0.109	4	82	48	106	0.1315	>1aa
185	!GST→GST	Fisher	4.0×10^−5^	0.00088	0.05	1	85	28	128	0.1034	>1aa
185		CMI	<10^−3^	<10^−3^							
268	*E*→!E	Fisher	0.00011	0.033	0.24	9	68	50	90	0.2586	1aa
268	!K→K	Fisher	0.00026	0.088	0.68	1	83	24	134	0.1286	1aa
268	*ES*→!ES	Fisher	4.7×10^−05^	0.00006	0.21	8	69	50	90	0.2294	>1aa
268	!KR→KR	Fisher	7.8×10^−05^	0.0011	0.06	1	83	27	131	0.1122	>1aa
268		CMI	<10^−3^	<10^−3^							
364	!AH→AH	Fisher	0.0049	0.052	0.16	2	82	21	133	0.2202	b12
364	*PS*→!PS	Fisher	0.0018	0.0085	0.13	2	84	24	134	0.1906	b12
**369**	***AP*** **→!AP**	**Fisher**	**0.0048**	**0.017**	**0.077**	**1**	**37**	**9**	**25**	**0.1368**	**b12**
**369**	**!ILQ→ILQ**	**Fisher**	**0.0013**	**0.047**	**0**	**0**	**37**	**8**	**24**	**0.0731**	**b12**
**461**	**!E→** ***E***	**Fisher**	**0.00026**	**0.045**	**8.77**	**12**	**60**	**3**	**133**	**7.1393**	**1aa**
**461**	**!EP→** ***EP***	**Fisher**	**4.5×10^−5^**	**0.0009**	**8.59**	**15**	**57**	**4**	**132**	**7.3379**	**>1aa**
651	*N*→!N	Fisher	0.0007	0.064	0.24	6	68	38	101	0.2653	1aa
651	!DIS→DIS	Fisher	0.0082	0.025	0.25	2	76	20	126	0.2356	>1aa
655		CMI	0.002	0.12							
149^9^	**Nx[ST]→** ***Nx[ST]***	Fisher	0.0044	0.22							PNLG
V2^10^	*Shorter length*	Spearman	0.021	0.08							
V5^10^	*Fewer PNLGs*	Spearman	0.0065	0.065							

1 **HXB2 position** refers to the amino acid position of interest in the HXB2 reference strain (www.hiv.lanl.gov: Locator tool).

2 **Amino acid** refers to the particular amino acid or combination of amino acids that was statistically related to b12 resistance (underlined) or sensitivity (not underlined and italics). An exclamation point means “not”; thus in the first line, when T is an ancestral state, Y mutates to “not Y” (!Y) with a statistically higher frequency in b12 resistant strains than sensitive strains.

3 **Statistic** is the statistic that was used to identify the signature, by either the phylogenetically corrected contingency approach (Fisher exact test) employed as described in [54]; the conditional mutual information approach (CMI); or a comparison of all variable region loop lengths (length) and number of glycosylation sites (sequons with amino acid pattern Nx[ST]) with the b12 neutralization values using a Spearman rank correlation test.

4 The **p-values**, the **q-values** (false discovery rates), and the **odds ratios** are provided. The Fisher's exact test q-values were calculated for discrete tests as implemented in [54]. For the CMI analyses, p-values were acquired by shuffling phenotypes and counting the relative frequency at which random CMIs exceeded the original CMI. The q-values were calculated using the method of [124], after stripping off the highest p values (essentially a few hundred of p-value = 1). Only associations with a q-value<0.2 are shown.

5 Rows and columns of the 2×2 contingency table. As an example of how to read these, in position 173, **r1c1** refers to row 1 column 1 and is the number of times among b12 sensitive viruses that Y→!Y mutates to another amino acid (change). **r1c2** refers to row 1 column 2, and it is the number of times among sensitive viruses that the ancestral state was Y and it stayed Y (stable) in the Env sequence.

6 **Strength** is a measure that expresses how predictive a given signature amino acid is of the b12 sensitive/resistant phenotype, essentially an augmented odds ratio, where each count was augmented by 1 pseudo-count to avoid issues with zeros and infinities, and strength = *(r1c1+1)(r2c2+1)/(r1c2+1)(r2c1+1)*.

7 Several explorations of the Env alignment were used, and this is described in the “**test**”. In our first screen, every amino acid found in every column was tested (1aa). Then combinations of 2 or more amino acids in every column were tested (>1aa). Then positions known to be key for the b12 binding site (Sup. Table S3) were specifically tested for all combinations of amino acids over all pairs of positions in the binding site (b12). Although pairs of positions were tested, single positions essentially accounted for the signal in that analysis. Only these single site associations are shown.

8 Some lines are shown in bold. In these lines, the change in the amino acid is associated with a reverse in the majority of cases found among sensitive or resistant viruses; thus the change in these sites is particularly predictive of NAb phenotype.

9 All PNLG sites in Env were tested for phylogenetically corrected association with b12 sensitivity using the contingency table approach. None reached significance with a q-value of <0.2; the glycosylation site at position 149 was the only one to reach even borderline significance and is included here for completeness.

10 For the initial analysis of loop length and number of PNLGs in each loop, we did not used a phylogenetically corrected method. Rather we used a non-parametric Spearman's correlation test comparing loop length with the geometric mean 50% neutralization titer for the 25 Envs. It is reasonable to forego the phylogenetic correction in these cases because the loop lengths vary by insertion and deletion and often change dramatically within infected individuals. These parameters are less likely to be biased by phylogeny at the population level.

A representative example of a single amino acid contingency analysis through the maximum likelihood tree, Aspartic Acid (D) at position 185, is illustrated in Fig. 1. The simple uncorrected Fisher's exact p value for this signature amino acid example (p<10^−8^) indicated that a D in position 185 is highly associated with b12 sensitivity. The low p-values for the patterns of change and stability relative to the most recent ancestral state as estimated through the maximum likelihood tree, showed that mutation away from D in resistant viruses (p = 0.0005), and towards D in sensitive viruses (p = 0.0004) were also associated with b12 sensitivity, providing assurance that the profound association with 185D and b12 sensitivity was not simply an artifact of shared lineages (Fig. 1, Tables 3 and 4). The low q values (Table 2, q = 0.06 and q = 0.04, respectively) indicate that these low p-values are not expected by chance alone, despite the very large number of tests performed (i.e., every amino acid found in every position in Env, and all combinations of amino acids in every position).

**Table 3:** Prediction strategies for b12 sensitivity applied to the 251 pseudotyped Envs included in the signature-defining training set.

Sensitive Envelopes:	Total	Correct	Incorrect	Sensitivity
Signature rule	88	67	21	0.76
Logistic regression	88	53	35	0.60
Ensemble Learning Technique	88	64	24	0.73

Sensitive Envelopes:	Total	Correct	Incorrect	Specificity
Signature rule	163	110	53	0.67
Logistic regression	163	134	29	0.82
Ensemble Learning Technique	163	153	10	0.94

Summary of all Envelopes:	Total	Correct	Accuracy	Fischer's p-value
Signature rule	251	177	0.71	2.4×10^−11^
Logistic regression	251	187	0.74	1.4×10^−11^
Ensemble Learning Technique	251	217	0.86	<2.2×10^−16^

**Table 4:** Prediction strategies for b12 sensitivity applied to the 56 pseudotyped Envs included in the blinded test set.

Sensitive Envelopes:	Total	Correct	Incorrect	Sensitivity
Signature rule	20	13	7	0.65
Logistic regression	20	9	11	0.45
Ensemble Learning Technique	20	5	15	0.25

Sensitive Envelopes:	Total	Correct	Incorrect	Specificity
Signature rule	36	26	10	0.72
Logistic regression	36	27	9	0.75
Ensemble Learning Technique	36	29	7	0.72

Summary of all Envelopes:	Total	Correct	Accuracy	Fischer's p-value
Signature rule	56	39	0.70	0.007
Logistic regression	56	36	0.64	0.11
Ensemble Learning Technique	56	34	0.61	0.44

We also analyzed all potential N-linked glycosylation sites (based on the presence or absence of the amino acid N-linked glycosylation motif NX[ST] in a give position in the Env alignment) for associations with b12 activity, again using a phylogenetic correction. None had a q-value<0.2, and the only one that showed borderline significance was found at position 149 (it is noted in Table 2). Finally, we also explored the b12/gp120 interface more deeply, including all combinations of amino acids in all pairs of sites in this region. Single sites accounted for most of the statistically significant signatures in the b12 binding region (Table 2). (A listing of the sites included in the b12 binding region is available in Table S3).

Of these 7 sites defined by phylogenetically corrected contingency analyses, 5 were also identified as b12 signatures by an ensemble learning technique using classification trees, while 3 were also identified by conditional mutual information (CMI) analysis (Table 1). The best predictors from the ensemble learning approach included a subset of the most significant amino acids in the contingency table (Tables 1 and 2), and did not add any new information. An additional 3 signature sites were uniquely identified by CMI analysis: 2 in gp120 and 1 in gp41 (Tables 1 and 2). The CMI approach was used to increase our sensitivity, and possibly to capture additional sites of interest. The contingency table analyses restrict each comparison at each site to a particular amino acid or combinations of amino acids in the ancestral states immediately preceding the endpoint taxa, thus using only a subset of the available data for statistical analysis. In contrast, CMI utilizes information across all possible ancestral states at the immediate ancestral node of the tip, but does not identify particular amino acids at the site of interest, just the sites that had mutational patterns associated with resistance or susceptibility. An alignment of the three additional sites that were identified by the CMI method is provided in supplement Fig. S1. Each of these positions was relatively conserved; examining these alignments suggests the consensus amino acids at the three sites, 163T, 182V, and 655K ,are well tolerated among viruses with b12 sensitivity, but that mutations 163A, 182E and mutations away from 655K, were enriched among resistant viruses.

**Figure 3:**
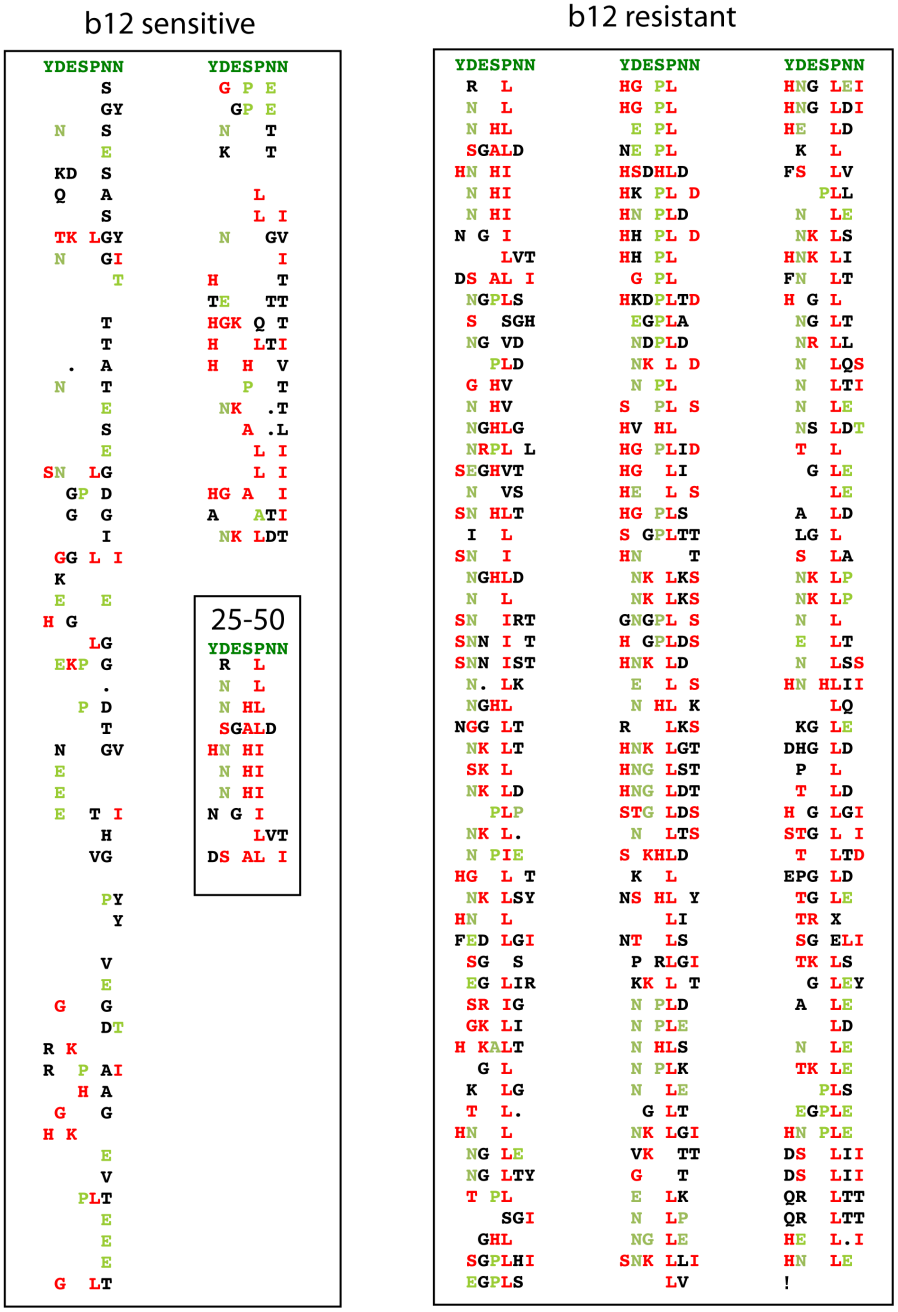
Alignment of b12 signature positions from each sequence with particular amino acids associated with b12 resistance and sensitivity. This alignment includes the 7 non-contiguous positions found using the contingency table approach with defined resistant/susceptibility patterns: positions 173, 185, 268, 364, 369, 461, and 651. The positions are aligned to the consensus of the susceptible viruses, which in each case is an amino acid that was associated with b12 susceptibility, shown in dark green at the top of each column. If the amino acid was the same as the b12 sensitive consensus at the top of the column, a space is left in the row, indicative of the consensus susceptible form. Amino acids that differed from the susceptible consensus, but were associated with susceptibility, are light green. Amino acids associated with resistance are red. Amino acids that were not associated with either resistance or susceptibility are black. The susceptible viruses are ordered from the top left column through the second column, from the most sensitive to the least sensitive in terms of the concentration required for 50% neutralization. The least sensitive Envs (those require concentrations of 25–50 µg/ml of b12) are boxed at the bottom of the second column.

It is important to remember that while these associations are statistically supported (Tables 1 and 2), any mutation in isolation may not be able to alter the phenotype of a virus in the context of a given natural strain. For example, although a change away from D at position 185 was most significantly associated with b12 resistance, and was most predictive of the phenotype, some Envs carrying the mutation remained b12 sensitive in 13/48 (27%) natural occurrences of this pattern. Thus, the signatures we identified point to the biological relevance of mutational patterns among a population of circulating viruses, but are not necessarily predictive in isolation in a single strain. Despite this, higher frequencies of amino acid substitutions associated with a b12 resistant phenotype, and loss of substitutions associated with a b12 sensitive phenotype, summed over all 7 signature sites, were strongly associated with resistance. This indicates that effects at the positions identified were cumulative. Notably, the signature sites were identified based on a simple Boolean resistant/sensitive phenotype, yet resistance-associated amino acids accumulated across these sites in viruses with diminishing b12 sensitivity. Specifically, the left hand box in Fig. 3 includes all b12 sensitive pseudoviruses tested, and is ordered by diminishing sensitivity. Combinations of more resistant and fewer sensitive amino acids are clearly evident among the least sensitive viruses nearing the end of the columns.

### Structural and biological interpretation of the b12 signature sites

#### b12 contact surface signatures in gp120

**Figure 4:**
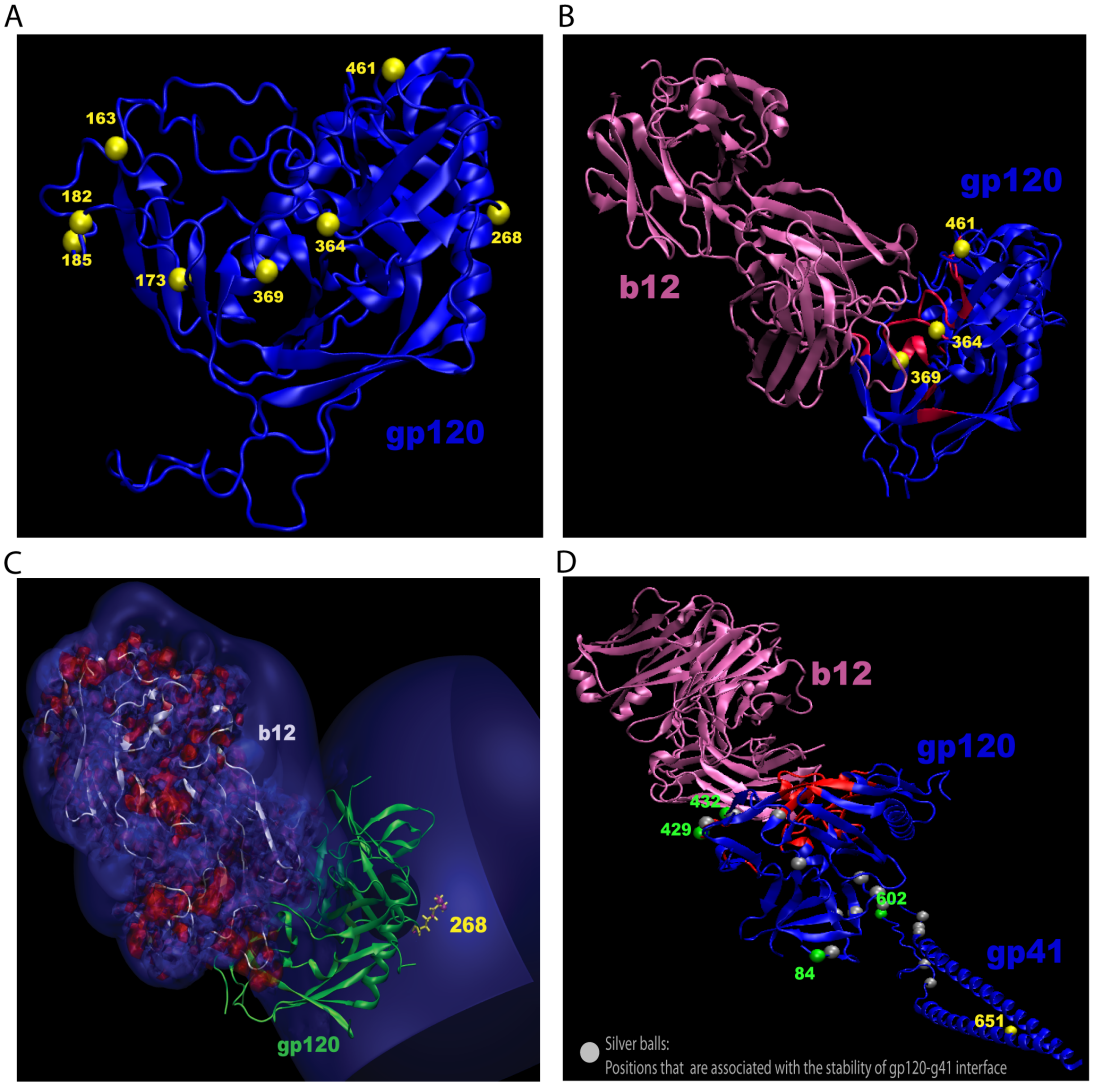
Structural mapping of b12 signature sites in gp120. (**A**) b12 signature sites in a three-dimensional structure of gp120 (PDB code: 1RZK) with V1, V2 and V3 loops modeled for visualization as described previously [128]. Yellow balls mark the C-alpha positions of signature residues. (**B**) Locations of 3 signature sites that occur at the b12 binding face of gp120. The b12 (magenta) bound structure of gp120 (blue), corresponding to PDB code:2NY7. The red region in gp120 is within 6.5 Å from the bound b12 antibody. (**C**) Isosurface of the gp120 molecule showing the difference in electrostatic potential (+0.3 kT/e) due to mutation E268R in gp120 that results in a net positive electrostatic potential (blue) at the b12-gp120 interface region. Isosurface (+/−1 kT/e ) of the b12 molecule showing the positive (blue) and negative (red) electrostatic potentials indicating b12 is highly electropositive (overall charge of +12). (**D**) An illustration of position 651 could impact binding to b12 through an allosteric pathway involving the gp120-gp41 interface. The X-ray structure of b12 (marked in magenta) bound to a liganded gp120 core protein (PDB code: 2NY7) with a monomer gp41 that was homology-modeled based on the NMR structure of SIV post-fusion gp41 conformation [129]. The region of gp120 in contact with b12 is marked in red. The disulfide bridged loop region of the gp41 molecule that is expected to interact with gp120 was placed in close proximity to the region where the N- and C-termini of gp120 come in close contact. This model is useful for illustration but does not represent the actual gp120-gp41 interaction, which is not yet resolved. A yellow ball indicates position 651 in gp41. Green balls are used to show the covarying sites at positions 84, 169, 429 and 432 in gp120, and position 602 in gp41. Silver balls in the model capture sites in gp120 and gp41 that have been shown through past experimental studies to influence gp120-gp41 assembly.

Fig. 4a shows the locations of the 8 gp120 signature sites we found in a three-dimensional structure of gp120 [35], [60]–[62]. Three b12 signatures (positions 364, 369 and 461) occurred in (364 and 369) or near (461) the b12 contact surface of gp120 [35], [58]. These three sites are shown in the context of a b12-bound gp120 structure in Fig. 4b. Sites 364 and 369 are located in the CD4 binding loop in the outer domain of gp120, where both sites directly contact residues in the heavy chain of b12 in a crystallographic structure of b12 Fab complexed with a stabilized gp120 core molecule [35], and mutations at these positions have been shown to alter the b12 susceptibility of multiple HIV-1 viruses [58], [63], [64]. Alanine scanning showed that an N to A substitution at position 461 could diminish b12 binding affinity more than 10-fold [64]. Because site 461contacts CD4 and lies adjacent to residues that directly contact b12 in the gp120-b12 crystal structure [35], it may affect epitope exposure.

Wu et al. identified 3 amino acid substitution patterns (S364H, P369L/T/Q and T373M) that were predicted to impact b12 binding because of potential clashes in side chain rotomers at the b12 contact surface [58]; two of these were among our signature sites (364 and 369). They showed that an S to H substitution at position 364 substantially increased b12 binding and neutralization susceptibility in several natural viruses. In contrast, the relevant substitutions at positions 369 and 373 impacted b12 binding to gp120, but did not restore neutralization to several resistant natural viruses, suggesting the epitope was shielded in the functional Env trimer in these strains [58]. Our analyses indicated that a P or S at position 364 was associated with susceptibility, whereas an A or H at this position was associated with resistance. In addition, an A or P at position 369 was associated with susceptibility, whereas an I, L, or Q was associated with resistance (Table 1). The T at position 369 that was predicted by Wu et al. [58] to interfere with binding is rare, and was found only once in our data; that single occurrence was in a susceptible virus (Fig. 3). The third site identified by Wu et al., mutation T373M, was not found among our signature sites. In Wu et al., 373M was enriched among subtype B resistant viruses in conjunction with other mutations. In our study, 34% of the b12-sensitive viruses overall carried an M at position 373, whereas 28% of the resistant viruses carried an M, thus no significant association was found between an M at position 373 and resistance, in fact M was slightly more common among sensitive viruses. There was a trend suggesting mutations away from T at position 373 were more common among resistant viruses (p = 0.057); however, this did not approach significance (q = 1).

#### V2 region b12 signatures

Four additional signatures (sites 163, 173, 182 and 185) occur near the C-terminus of the V2 region of gp120 (Fig. 4a). Some regions of V2 are hypervariable and contain frequent insertions and deletions, but alignment positions that span these regions are excluded from the signatures analysis at the outset because positions that contain more than 10% gaps are not reliably alignable, so not tested for signatures here (see Materials and Methods section). So while insertion and deletion patterns in V2 may impact b12 binding, we are only able to identify amino acid signatures in V2 that were embedded in regions of V2 that are conserved enough to be readily aligned with our analysis. Because no X-ray crystal structures of gp120 are available with an intact V2 loop, the positions on the loop are shown on a modeled loop for visualization (Fig. 4a, see Materials and Methods section). Based on the crystal structure of the V1/V2 stem, positions near the C-terminal end of the V2 loop are predicted to impact the b12 epitope [61], [62]. Indeed, results from Alanine scanning mutagenesis confirm the critical importance of the V2 region for b12 binding. For example, a D to A substitution at our signature position 185 was previously found to diminish b12 binding affinity greater than 10-fold [64]. Moreover, a mutation in this position resulted in escape from b12 neutralization [63]. We also found that significantly reduced V2 loop lengths, and a reduced number of potential N-linked glycosylation sites in the V5 loop, were associated with b12 neutralization (Table 2). A complete scan of the gain or loss of individual PNLGs throughout Env did not reveal an association with any one particular glycosylation site in b12 binding at the statistical threshold of q<0.2.

#### The b12 signature at site 268

To our knowledge, site 268 has not been previously investigated for an effect on b12 binding and neutralizing activity. This site is spatially distant from the interface of b12 and gp120, located approximately 30 Å away [35] (Fig. 4a). Intriguingly, this signature involved a charge reversal from an acidic residue to a basic residue resulting in a +2 charge change at this site. Such a change could potentially have a long-range electrostatic effect, thereby impacting b12 binding, particularly since b12 itself is highly positively charged. Therefore we carried out electrostatic potential calculations using the Adaptive Poisson-Boltzmann Solver (APBS) to quantify the change in electrostatic contributions to the b12 binding arising from the substitution of a negative with a positive charge at this position. APBS solves the Poisson-Boltzmann equation, a continuum model for describing electrostatic interactions numerically [65]. We used the recent X-ray structure of b12-bound to the JRFL gp120 for these calculations [35], and modeled the appropriate site-mutations in the backbone of JRFL. The overall structure was not relaxed and only the side-chain rotomer of the replaced residue was positioned in an energetically feasible position. We found that a change from 268E to either 268R or K results in an estimated decrease of b12 binding by 1.4 Kcal/mol. In Fig. 4c, the isosurface surrounding gp120 shows the difference in electrostatic potential (+0.3 kT/e) due to the mutation E268R on gp120; interestingly the isosurface is close to the b12-gp120 interface region. This figure also shows that b12 is highly electropositive (isosurface of +/−1 kT/e) due to the charged nature of b12 (overall charge of +12), explaining the large decrease in binding energy upon E268R mutation. This is consistent with the phenotypic directionality captured by the signature analysis.

Our finding of a b12 signature at site 268 that underwent a charge reversal prompted us to explore whether there are additional acidic residues in gp120 that could undergo similar charge changes. Obviously not all charged residues are in a position to reverse their charge state to escape immune pressure. Some are highly conserved due to functional constraints. Other acidic residues may take part in critical electrostatic interactions that stabilize the structure. Often charged residues are involved in salt-bridge interactions. In this latter case it is possible that co-varying charge changes could occur simultaneously at the salt bridge forming partners (i.e.: K/R—E/D salt bridge pair becomes E/D—K/R pair); a simple continuum electrostatics model would then predict no significant effect on electrostatic binding energy. To address these possibilities, we systematically examined all of the acidic residues in the gp120 in the gp120-b12 bound X-ray structure. Details of these sites are provided in Table S4. Except for positions 106 and 268, all other positions had dependencies that prevent a negative to positive change, either due to salt-bridge interactions or sequence conservation. Positions 106 and 268 are the only acidic residues in the gp120 core that are not conserved and do not take part in a salt bridge interaction. Thus, site 268 may provide a rare opportunity for a charge reversal pathway that would allow the virus to become resistant to neutralization by b12 or other positively charged antibodies.

#### b12 signatures in gp41

Two statistically significant signatures were identified in gp41. Both sites (positions 651 and 655) are in the C-heptad repeat, which is expected to lie proximal to the N-heptad repeat targeted by the HIV-1 fusion inhibitor T-20 in the post-fusion conformation [66]. The C-heptad repeat also contributes to the formation of a six-helix bundle that mediates viral fusion with the cellular membrane [67]. Finding b12 signatures in gp41 is not unexpected, as mutations in gp41 are known to affect NAb epitopes in the CD4bs [68]–[76], including the b12 epitope [58], [69]. These mutations include amino acids at positions 569, 577, 582, 668 and 675 in gp41 that affect CD4bs epitopes; and mutations at positions 569 and 675 affect the b12 epitope directly [58], [69]. While positions 651 and 655 have not been directly implicated in b12 binding in previous studies, those studies were based on escape mutations in single virus strains (IIIB, MN, JR-CSF, Q461, Q769, YU-2). In contrast, our study was based on systematically identifying significant associations among 251 genetically diverse viruses. This broader scope of analysis may have led to the identification of sites in gp41 that more generally affect the b12 epitope among global variants.

To explore the question of how sites in the gp41 C-heptad repeat that are distant from the gp120-b12 binding interface could influence the b12 epitope, we began by identifying all sites within Env that significantly co-vary (hence potentially interact) with positions 655 and 651. To do this, we used the phylogenetically corrected contingency table approach to identify the sites that covaried with signature sites in Envelope. The resulting co-variation patterns for all 10 of our b12 signature sites, including the two gp41 signature sites, are summarized in Table S5. Position 655 was found to significantly co-vary with a single position, site 185, which was also the most significant signature site in gp120. As noted above, this site is located in the V2 region of gp120 and has been shown to be a critical residue for b12 binding affinity [64]. Thus, the association between mutational patterns in position 655 and b12 neutralization could be a consequence of quaternary structural interactions, giving rise directly to the correlation between mutational patterns of position 655 and b12 sensitivity. Alternatively, the 185–655 interactions could be driven by a relationship that is independent of the b12 epitope. In this latter case, the statistical association between site 655 and b12 neutralization may be due to a correlation that is one step removed, i.e. an ancillary consequence of the direct interactions of site 185 and b12. 655K is the most common amino acid in this position, where both K and E appear to be associated with b12 neutralization sensitivity in our signature analysis. As an aside, O'Rouke et al. [77] studied in detail the impact of substitutions on neutralization in a site they call 655, but because they did not use standard HXB2 numbering, their site 655 is actually 653 in HXB2, and so is not the signature site identified here.

Covariation patterns were more complex for site 651, which was found to have 9 covarying sites (Table S5), 4 of which are captured in a schematic molecular diagram in Fig. 4d. Site 80 and site 169 are in a region of the V2 loop for which no crystal structure is available and therefore were excluded from gp120 in this diagram. Similarly, 3 sites were in the cytoplasmic tail and thus were not included here (sites 798, 817, and 822). Based on crystallographic data, covarying sites 429 and 432 (though not statistically supported b12 signatures) are spatially close to the CD4 binding loop in a region that contacts b12 [35]. A K432A substitution diminished b12 binding affinity >10-fold [64]. The presence of this complex chain of covarying sites in gp41 and gp120 suggests allosteric effects, where site 651 is part of a set of spatially distant residues that modulate the gp120-gp41 interface and thereby influence the exposure of the b12 epitope in the quaternary configuration of Env. Receptor and coreceptor binding induce structural re-arrangements at the gp120-gp41 interface as a requisite step for membrane fusion [18], [20]. In principle, genetic changes that influence the gp120-gp41 contact surface could have reciprocal allosteric effects on the CD4bs of gp120. Consistent with this hypothesis, two of the 651 covarying sites (position 84 in the N-terminal C1 region of gp120; position 602 in the gp41 disulfide loop) occur in regions implicated directly in gp120-gp41 contact and stability [78]–[85] (Fig. 4d). Alternatively, the mutations in site 651 that correlate with b12 susceptibility might influence a different allosteric pathway that relies on quaternary interactions with the CD4 binding loop region (sites 429 and 432) or possibly V2 (site 169) in the context of a trimer.

#### Predictions of b12 neutralization

Our primary purpose in this study was the identification of signature sties discussed above, but we used three computational approaches (described in the Materials and Methods section) to determine if we could predict b12 neutralization phenotypes based on sequence information. Prediction strategies were developing based on the “training” set of 251 sequences used to define the original signature pattern. The three strategies were tested by predicting the b12 phenotype in a blinded set of 56 pseudotyped Env sequences. The first two tests were implemented essentially to explore the potential of the specific signature amino acids defined at the seven positions by correlation analysis to prediction of phenotype on a blinded set of data, essentially as a further test of their relevance to b12. The third test was a classic machine learning test based on all sites in Env. The first strategy applied a simple rule based on inspection of the alignment of the seven signature sites with defined amino acids shown in Fig. 3. If the sequences contained at least 4 “sensitive” amino acids, and no more than 1 resistant amino acid in these seven sites, we classified it as sensitive. In our second approach, we used logistic regression to formalize the contribution of change at each site in an attempt to refine the predictive ability of the signature. Our third approach was to apply an ensemble learning technique using classification trees to the amino acid changes in the full alignment, with the thought that this method could be used both for prediction of b12 phenotype based on the full Env sequence, and for defining the particular signature positions and amino acids which contributed most to the b12 phenotype (Table 1). When applying the three methods to the original training set 251 viruses, we found that the simple rule based on the alignment was less predictive than the logistic regression, and the ensemble learning method was the most predictive (Table 3). When we applied the three methods to the blinded test set, however, the order reversed, and in this case the first simple method was the most predictive (p-value = 0.007, Table 4). The predictive power of this simple signature based strategy further supports the relevance of the b12 signature sites and amino acids associations. The other two methods had higher rates of false negatives and were not significantly predictive (Table 4). Reason for this inadequate power is not clear, but could be due to differences in the sampling of the 251 viruses and the 56 viruses that limited the predictive power of the two computational prediction methods. The full set of predictions based on the three methods and the b12 experimental data are provided in Table S2.

As discussed previously, the signature sites were originally defined based on a simple classification of b12 sensitive or resistant phenotype. Thus, as seen in the left hand panel in Fig. 3, the cumulative number of sensitive amino acids in the 7 positions tends to decrease as b12 sensitivity diminishes (green amino acids and agreement with the most common sensitive form), whereas resistant amino acids tend to accumulate (red amino acids). To formally test whether *level* of b12 sensitivity among the sensitive viruses was correlated with the signature pattern, we first reduced the signature pattern to a single sensitivity score, obtained by subtracting the number of resistant amino acids from sensitive amino acids (red from green, in Fig. 3). The signature sensitivity score was correlated with b12 sensitivity (p = 0.0006, Spearman's rho = −0.34, Fig. S2). Thus signature amino acids can be used to predict, with significant accuracy, both the initial sensitive and resistant classification and the level of sensitivity among b12 sensitive viruses. Because these sites were identified after correcting for founder effects in the training set, we may assume that the correlation observed is causal.

### Signature analysis of Envs that elicit potent NAb responses in HIV-1-infected individuals

#### Clustering sera according to cross-reactivity and potency

**Figure 5:**
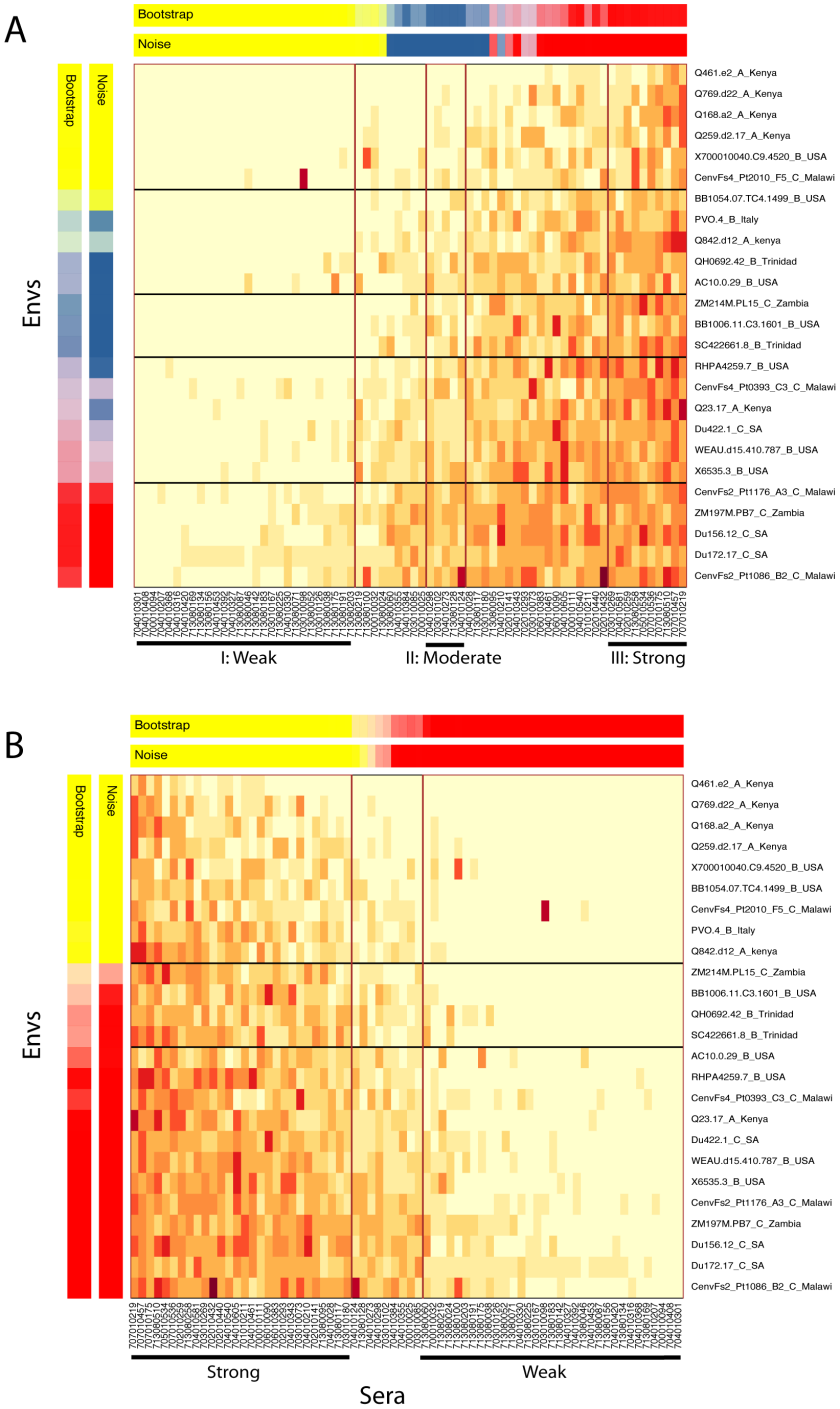
Clustered heatmaps of sera and the test panel. (**A**) K-means clustering of serum samples and virus isolates in the test panel, k = 3. A 90% threshold for stability was used as a minimum criterion for defining robust clusters in the sera, given re-sampling noise due to experimental variation and bootstrap re-sampling of the test panel of Envs. 75% was used for the clustering the Envs in the figure, and these clusters were not subsequently used for analysis. The color keys on the top and on the left indicate the clusters and their statistical robustness: red, blue and yellow correspond to the three clusters, with each robust cluster boxed. Blends of the three primary colors indicate how often in the re-sampling tests for a given serum or Env the sample falls in different clusters, and the intensity of the color indicates how frequently each falls in its primary cluster. Within the heat map, darker red indicates potent neutralization, progressively lighter colors through yellow indicate increasing resistance, and cream color is completely resistant. (**B**) K-means clustering of serum samples and virus isolates in the test panel, k = 2; again a 90% threshold for stability was used for the sera, 75% for the viral Envs.

**Figure 6:**
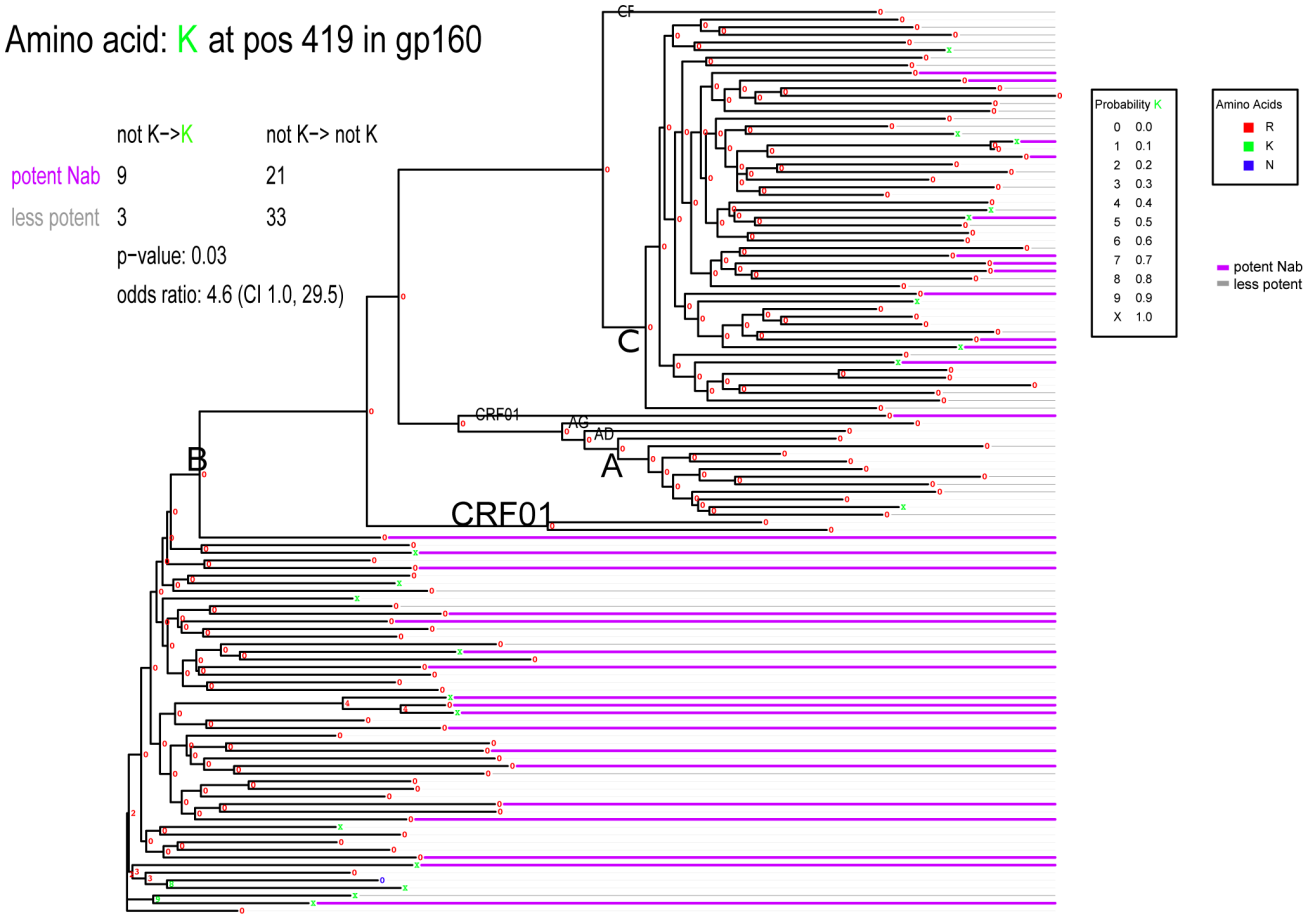
Maximum Likelihood tree of the Env sequences showing ancestral states and amino acid in the end taxa for position 185. This tree illustrates the phylogenetic distribution of Envs sampled from individuals with potently neutralizing natural antibody responses; magneta lines indicating the Envs taken from individuals with potent sera, and gray lines, weak sera. The taxa without a magenta or dark gray line were from the test panel of psudotyped Envs. The evolution of the signature site 419 K with respect to the phylogenetic tree is highlighted. Arg (R) is the most common amino acid in this position, and K is very rarely an ancestral state.

We next sought to determine whether our signature analyses methods could be used to identify amino acids that associate Envs able to induce with broadly cross-reactive NAb responses in HIV-1-infected individuals, where the NAb responses were clustered according to potency using K-means (Fig. 5). Env sequences and neutralizing activities in sera from 69 chronically infected individuals were used for analyses. The serum samples were obtained from individuals in the United States, Malawi, South Africa, Tanzania and England and consisted of: 1 CF recombinant, 1 CRF01_AE, 1 A/G recombinant, 5 subtype A, 24 subtype B, and 37 subtype C HIV-1 infections (Fig. 6, and Table S6 in the supplemental material). These 69 serum samples were chosen from among 360 sera that were assayed against an initial screening panel of twelve viruses (6535.3, QH0692.42, SC422661.8, PVO.4, AC10.0.29, RHPA4259.7, Du156.12, Du172.17, Du422.1, ZM197M.PB7, ZM214M.PL15, CAP45.2.00.G3). The 69 selected samples represented a wide spectrum of neutralization potencies against these 12 viruses. For increased statistical power in terms of robust assignments of potent versus weakly cross-neutralizing sera, the 69 sera were assayed against an additional multi-subtype panel of viruses, such that the total number of pseudoviruses assayed was 25 (6 subtype A, 10 subtype B, 8 C and 1 BC recombinant, all isolated early in infection, see Table S7 supplemental material). The final checkerboard-style results (Fig. 5) confirmed a wide spectrum of neutralization potencies, including a subset of samples that contained high titers of NAbs against a majority of viruses tested, and for contrast, a subset of comparable size that was poorly cross-neutralizing.

The combined neutralization results were clustered according to the ability of individual serum samples to neutralize the panel of 25 viruses, using a k-means strategy that factors in the robustness of the clusters according to the uncertainty that results from limiting sampling (bootstrap) and assay-to-assay variability (noise) (Fig. 5). To assess the impact of assay variability, we factored in error estimates based on a limited number of repeat experiments. To do this we added back error (drawn from a log-normal distribution based on the repeat data) to the real data, and created 1000 reconstructed data sets. This allowed us to resolve clusters that should be robust relative to inter-assay variation (Fig. 5, noise). Next, we re-sampled from among the 25 Envs used in the neutralization assays 1000 times to see if the clusters would be robust if we had selected a different test panel of Envs with similar, but less diverse, composition. Fig. 5A shows 3 distinct clusters (k = 3) that turned out to include sera with high, medium and low neutralization potencies, respectively. k = 3 was the maximum number of clusters that could be meaningfully assigned, given the constraint that each cluster must contain at least 2 members, and that the members must meet the stability criteria of being associated with the assigned cluster in >90% of each of the two re-samplings experiments (i.e., “bootstrap” and “noise”). Standard k-means strategy was used to assign each serum to a single k-cluster; however, if based on re-sampling statistics described above, some sera could not be assigned to any of the k = 3 clusters, they are shown as intermediate values. The sets of sera that were grouped into each of the clusters, as well as those sera that are considered intermediate, are shown in Fig. 5. To make use of all 69 data points in a Boolean framework for signature analysis, including these intermediate values, three sets of signature analyses were conducted for the k = 3 clusters, comparing each one of the 3 robust clusters to all other data points. We also performed a k = 2 clustering that enabled a robust extreme “high” and “low” 2-cluster comparison that captured most of the data (Fig. 5B). This latter signature analysis did not resolve new signature site, but did sometimes improve the statistical confidence in a given site (Tables 5 and 6). Phylogenetically corrected methods similar to those described for the b12 sensitivity signatures were used to identify associations between serum Env sequences and distinct neutralization clusters.

**Table 5:** Sites identified as Env signatures associated with serum neutralizing breadth and potency using the tree corrected contingency table and CMI approaches.

HXB2 Position	Signature region^1^	CMI	Fisher's^2^ Sensitive/Resistant 2
412/413	CoRbs	-	![GS]N→[GS]N
413	CoRbs	-	Nx[ST]
419_421	CoRbs	-	R_K→!R_K
419	CoRbs	-	R→K
440	CoRbs	-	Q→!Q
186	V2	Y	

This table is organized similarly to Table 1.

1 All regions are in gp120. CoRbs, coreceptor binding site; V2, second variable region.

2 Arrows are used to show the direction of the sequence change that was significant. Thus, in the three amino acids at positions 419–421, the sequence was moving from R, any amino acid, K (R_K) to a sequence that was not R, any amino acid, K (!R_K) in weakly neutralizing sera. [GS]N, means either G or S at position 412 and N at 413.

**Table 6:** Summary of statistics of signature sites of associated with serum neutralizing breadth and potency.

HXB2 Position	Amino Acid	Statistic	p-value	q-value	Odds ratio	r1c1 Sensitive Change	r1c2 Sensitive Stable	r2c1 Resistant Change	r2c2Resistant Stable	Strength	Test^2^
**412/413**	**![GS]N**→***[GS]N***	**Fisher**	**2.1×10^−6^**	**0.0015**	**Infinity**	**6**	**4**	**0**	**57**	**81.2**	**2 sites, Full Env** **2 deep, k = 3, high vs others**
**413**	***Nx[ST]***→**!Nx[ST]**	**Fisher**	**0.0083**	**0.23**	**10.17**	**5**	**2**	**10**	**43**	**8**	**PNLGs, Full Env, k = 3, high vs other**
**419_421**	**R_K**→**!R_K**	**Fisher**	**0.0025**	**0.089**	**9.2**	**13**	**17**	**2**	**25**	**6.7407**	**3 sites, 2 deep** **CoRbs k = 3, low vs others**
**419_421**	**R_K**→**!R_K**	**Fisher**	**0.0013**		**8.1**	**13**	**17**	**3**	**33**	**6.6111**	**3 sites, 2 deep**CoRbs, **k = 2** **low vs high**
419	*R*→!R	Fisher	0.044	0.11	5.2	9	21	2	25	3.9394	1 site, CoRbs, k = 3, low vs others
419	!K→K	Fisher	0.044	0.11	5.2	9	21	2	25	3.9394	1 site, CoRbs, k = 3, low vs others
440	Q→!Q	Fisher	0.018	0.11	0	0	5	3	0	0.0417	3 sites, CoRbs, k = 3, low vs others
440	Q→!Q	Fisher	0.012	0.13	Infinity	3	0	0	6		3 sites, CoRbs, k = 2, high vs low
186		CMI	<0.001	<0.001							
V2	*Shorter length*	Spearman	0.043	0.14							
V2	*Fewer PNLGs*	Spearman	0.017	0.043							
V2	*Fewer PNLGs*	1Contrasts	0.06	0.06							
V5	*Shorter length*	1Contrasts	0.02	0.02							

This table is organized similarly to Table 2.

1 Variable loop lengths and the number of glycosylations sites in each variable loop were compared as in Table 1B, using a simple Spearman's rho test. We validated these results using a phylogenetically corrected method, phylogenetic contrasts [120], [121].

2 In this column the number of “sites” refers to the number of sites considered in combination in each test, the number “deep” refers to how amino acids at a single site were combined in each test. k = 2 or 3 refers to the k-means clusters as illustrated in Fig. 5. When k = 3, the test could either be the lowest or the highest neutralization potency cluster versus all others. When k = 2, only the high and the low clusters were compared, excluding indeterminate values. Full Env means the complete Env was scanned in the test. CoRbs means the signature was defined in the in-depth scan of the CoRbs. No significant signatures were found in comparable in-depth scans of the CD4bs and the MPER regions.

#### Defining signature patterns in serum-derived Envs

Envs sequences from all 69 sera were scanned for patterns of mutations that correlated with particularly weak or strong neutralizing capacities. Our analysis compared all single sites and all pairs of adjacent sites for signatures of either 1 amino acid or combinations of amino acids at each site. In this complete Env scan, a single signature was found in the CoRbs. This signature consisted of a pair of amino acids in which the combination of either G or S at position 412, together with N at 413, was found to be enriched in Envs from potent neutralizing sera. We then looked for signatures in potential N-linked glycosylation sites (PNLGs) throughout Env and again found a single signature pattern with borderline significance that was also located at position 413 in the CoRbs; in this case the PNLG was preserved in Envs from individuals with potent sera. Using the CMI approach to scan the full Env protein, an additional signature was identified at position 186 in the V2 loop.

**Figure 7:**
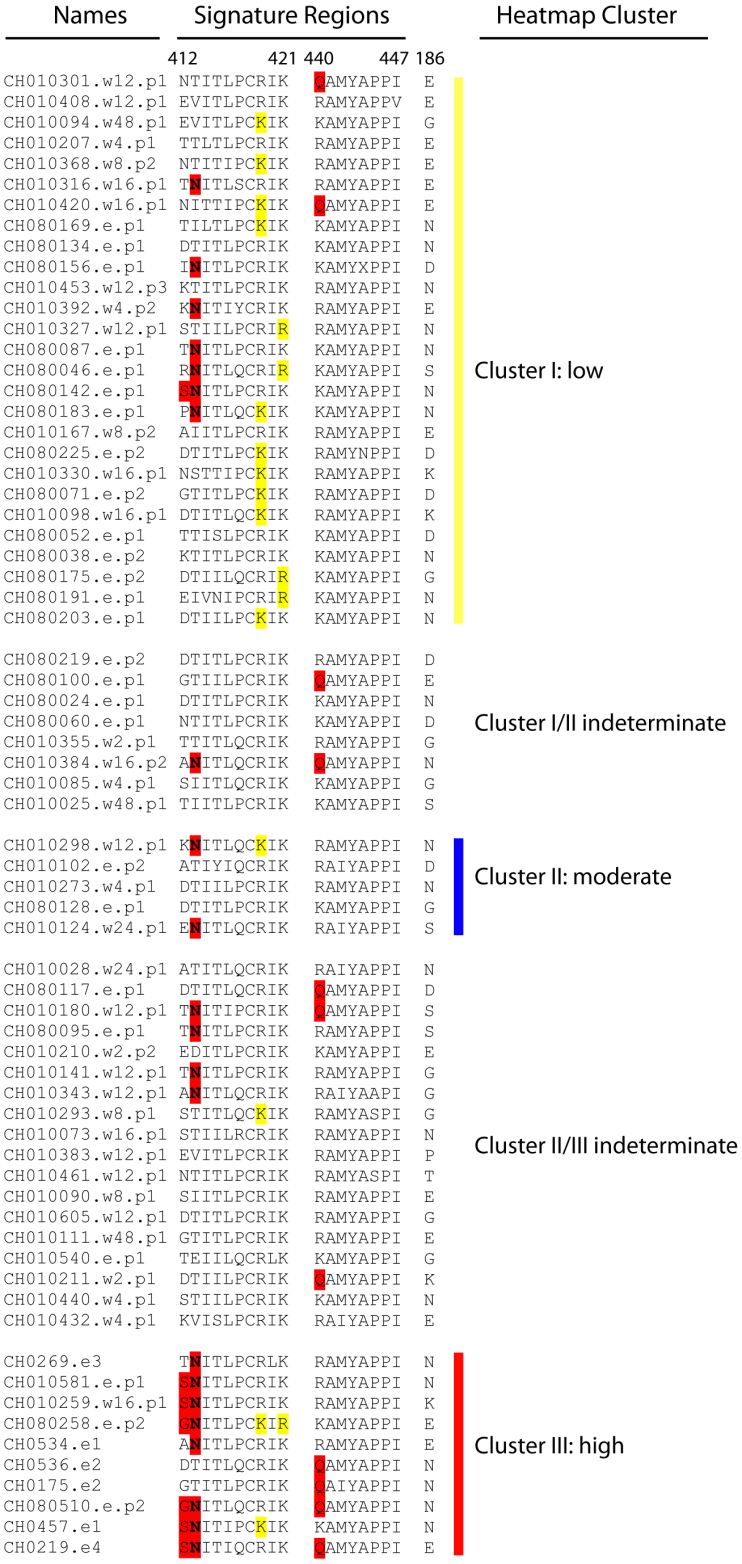
Alignment of signature sites that were associated with potent NAb responses. This alignment captures short contiguous regions of Env near the CoRbs. Signature amino acids are highlighted using the same color scheme and organization as the heatmap in Fig. 5A. Red highlights are amino acids that associate with potent sera; yellow highlights are amino acids that associate with weak sera. A vaccine strain selected on the basis of Envs in potent neutralizing sera from HIV-1-infected individuals might ideally capture as many of the red positions and as few of the yellow as possible (e.g., CH0219.e4 and CH080510.e.p2). CH0219.e4 also has short variable loops (data not shown). Position 186 was identified using CMI and thus does not have specific amino acids associated with the serological behavior; however, both E and N seem particularly enriched in the group with the highest cross-reactivity (cluster III).

We next performed a more in-depth exploration of regions in the CD4 binding site and CoRbs of gp120, and in the MPER of gp41. The sets of positions used for these analyses, and the references from which they were drawn, are listed in Table S3. These three regions were selected because antibodies against each one have each been identified in a subset of HIV infected people who possess potent cross-reactive NAb responses [86]–[89]. We examined combinations of multiple amino acids at multiple positions in these regions of interest. This sort of in-depth exploration was neither computationally feasible with the full Env, nor was it desirable because multiple test issues would have limited the power to find weak signatures if the full Env was explored so intensively. The deeper focused analysis revealed additional signatures, but only in the CoRbs (See Tables 5 and 6 for a complete summary of significant sites and their levels of significance). No correlations were found in either the CD4bs or MPER region even through these regions were also targeted for a more focused and in-depth analyses. Finally, as with b12 neutralization, we examined whether potent NAb responses were associated with other general features of Env that are known to affect epitope exposure, such as the number of PNLGs and the length of the hypervariable variable regions of gp120 [90]–[93]. The V2 loop was shorter with fewer PNLGs in Envs from subjects with potent sera, and Envs with shorter V5 loops [94] were also correlated with potent sera. The mutational patterns in all of the signature sites are highlighted in sub-region alignments in Fig. 7, ordered and colored according to the k = 3 heatmap clustering scheme shown in Fig. 5a. An Env such as CH0219.e4 might be particularly promising as a vaccine antigen, because it retains the full amino acid signature associated with potent antibody responses (Fig. 7), and it also has short variable loops (data not shown).

### Structural and biological interpretation of signature sites that correlated with potent NAb sera

**Figure 8:**
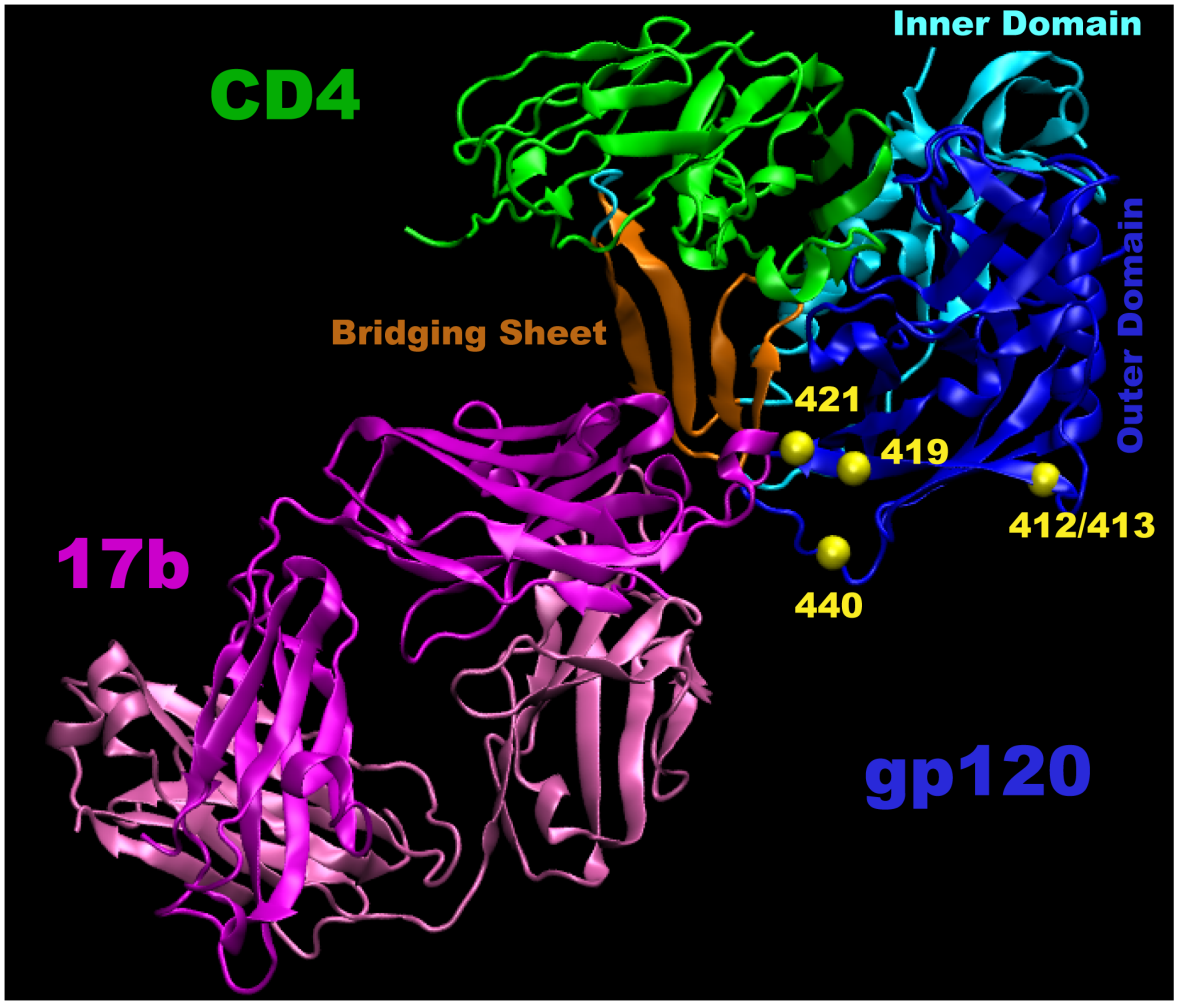
The four signature sites in the CCR5 CoR region shown in a crystallographic three-dimensional structure of gp120 complexed with CD4 and the CD4i-specific mAb 17b (PDB code: 1RZK). The yellow balls mark the C-alpha positions of the signature residues. Three regions in gp120 are indicated: the inner domain in light blue, the outer domain, dark blue; and the bridging sheet, brown. Definitions for these regions are based on the X-ray study of Kwong et al. [127]. CD4 is green. The light and heavy chains of 17b are marked in light and dark magenta, respectively.

The combined results of the contingency table signature analyses identified five statistically significant signature sites that resided in, or proximal to, the CCR5 CoRbs of gp120 (Tables 5 and 6). These sites are shown in a crystallographic model of gp120 complexed with CD4 and the CD4i-specific mAb 17b in Fig. 8. Sites 419 and 421 are located in the V4 region of gp120, immediately adjacent to the β20 strand of the bridging sheet that connects the inner and outer domains of gp120 [35], [61]. Both sites make contact with the CD4i-specific mAb 17b [61] (Fig. 8) and have been shown to be critical for CCR5 co-receptor binding [95]–[98]. Site 419 also makes contact with b12 [35], whereas site 421 is involved in the binding of other CD4i-specific mAbs E51 [98] and 48d [99] as well. Sites 413 and 440 in V4 and C5, respectively, are spatially close to the bridging sheet and overlap the contact surface for 17b [61]. Site 440 has been shown to be critical for CCR5 binding [96]–[98]. CMI analysis identified an additional site in the V2 loop, position 186, immediately adjacent to the b12 signature site at position 185. In addition to the position-based signature analysis, we found that strong NAb responses were associated with serum Env proteins that had fewer PNLGs and shorter lengths in V2 (Table 6). It has been shown that V1/V2 stem region can impact CCR5 binding since it plays a significant role in formation of the bridging sheet [96], [97]. Furthermore, site-directed mutational studies have shown that regions outside V3 loop, including site 166 (a position within V2 loop) can play a significant role in co-receptor usage/switch [94], [100]. Considering the flexibility of the loop and ensuing conformational changes that take place involving V1/V2 upon CD4 binding, a position such as 186 can directly or indirectly interact with critical sites involve in the formation of bridging sheet. The fact that no other signatures were identified suggests that the CCR5 CoRbs may play a substantial and relatively consistent role in the NAb response in HIV-1-infected individuals.

## Discussion

Assay technologies that utilize molecularly cloned Env-pseudotyped viruses with a defined sequence provide powerful tools for dissecting molecular determinants of neutralization epitopes on HIV-1. In addition to enabling mutagenesis studies, data from assays with clonal Env-pseudotyped viruses have been used for computational analysis to identify Env amino acid signatures that associate with the antigenic recognition patterns of autologous [53] and heterologous [52] NAbs in sera from HIV-1-infected individuals; such signatures could be contact sites for NAbs, or they may be determinants of epitope exposure in the quaternary structure of Env spikes.

Here we obtained partial validation of a computational strategy to identify amino acid positions that are related to NAb phenotypes. We systematically studied patterns of mutations in Env proteins that correlate with b12 susceptibility, and our signature analysis successfully identified key positions that are known from crystallographic and mutagenesis studies to be critical sites in the b12 epitope. Thus, 7/10 signature sites were identified either directly in the contact surface for b12, or in V2, which is known to impact b12 binding and neutralization. Notably, mutations in position 185 in V2 were nearly equal in strength to mutations in position 461, which were in this study the two best predictors for assessing b12 neutralization susceptibility in natural strains. Three novel positions were implicated in b12 neutralization based on amino acid associations. One was in gp120, at position 268; this signature raised a plausible hypothesis regarding the impact of electrostatic potential at the isosurface of gp120 on interactions with the positively charged b12 antibody. Two additional b12 signatures were identified in gp41 that were intriguing because they may affect exposure of the b12 epitope in the quaternary structure of Env. Interestingly, both sites in gp41 directly co-varied with sites at the b12-gp120 interface. Site-specific mutagenesis studies have been initiated to explore the impact of positions 268 and 651 on b12 neutralization, and the predicted signature substitutions at these positions were indeed found to capable of significantly impact b12 binding (manuscript in preparation). Two of the ten sites identified are statistically expected to be false positives, so it is likely that two will be not be found to be relevant when experimentally tested, although each of the ten sites and amino acids associations are biologically plausible, and most already validated in the experimental literature, as discussed in the results. Thus the b12 analysis provided a validation of using this approach for identifying signature patterns related to neutralization phenotypes, and provided new information by defining the particular mutations in the natural virus population that correlate with b12 neutralization susceptibility, and by determining the relative strength of such associations (Table 2).

The apparent accuracy of the b12 susceptibility signature analysis was encouraging; however, our findings highlight both limitations and virtues of these methods. Sequence-based signatures methods cannot be expected to identify all b12 contact residues in gp120 [35]; this is because some of these sites are highly conserved, whereas other sites at the contact interface may have natural variation that is well tolerated by b12. Yet other important sites might reside in hypervariable regions that cannot be aligned with confidence, so were excluded from our analysis – we attempted to examine the impact of these regions based on loop lengths and total number of glycosylation sites, which are alignment independent measures. In addition, since these methods scan full Env and started with no biological priors, they necessarily are based on a large number of tests that makes detecting weak signatures prohibitively data intensive. For example, we did not identify two PNLGs known to affect b12 susceptibility [58]. One of these sites was at the base the V2 loop (position 197) and the other was in the V3 loop (position 301). The PNLG in position 197 is almost invariant, and so could not have been identified by our method, which relies on detecting associations in the context of natural sequence variability. Position 301 (PNLG) reached borderline significance in the complete scan of Env when testing for an association between the preservation or loss of specific PNLGs and the b12 neutralization (p = 0.019, q = 0.30, OR = 0.23).

Signature methods focus on sites that are likely to be the most impacted by common mutational patterns found in the circulating population. Such mutational patterns are directly relevant for vaccine design considerations because we must contend with the natural variation of HIV for a vaccine to succeed. Indeed, signature methods provide a useful counterpoint to crystallography, which identifies the contact surface of a protein bound by antibody, but does not provide direct information about the implications of key common natural mutations [35]. Moreover, alanine scanning [64], which explores the functional impact of mutations introduced in either conserved or variable positions, is another extremely valuable tool, but one that is limited in terms of being able to look at the consequences of natural variation at specific sites or in combinations of sites. An additional limitation is experimental, in that some sites might require concentrations of b12 that are higher than those used here for positive identification. Despite these limitations, our computational analysis appears useful for delineating molecular determinants of complex neutralization epitopes on HIV-1 Env, including the identification distant sites that may impact b12 binding though quaternary and allosteric effects. The neutralizing impact of b12 is very specific, where slight differences in recognition sites between viruses can have major phenotypic consequences [101]. A better understanding of the impact of common natural mutations that are outside of the immediate binding surface of b12 may ultimately allow improved rational design strategies of vaccines that attempt to elicit potent anti-CD4bs antibodies.

Having confirmed that our computational analysis has utility for identifying molecular determinants of Env antigenicity in the context of the b12 epitope, we then sought to determine whether a similar computational analysis, but this time based on Env sequences derived from serum samples from HIV-1-infected individuals, could identify amino acid signatures that associate with the magnitude and breadth of the neutralizing activity of the serum samples. Any signatures identified by such analysis might be determinants of the immunogenic as well as antigenic properties of Env, although it was beyond the scope of this study to discriminate between these two immunologic properties.

For the analyses of Env sequences in serum samples that were evaluated for neutralizing activity, a single Env sequence from each individual was obtained. We were interested in leveraging our resources to increase the number of individuals studied rather than increasing the depth of characterization of each infected individual. In part we were testing the feasibility of the approach for scanning a large population of HIV infected individuals with the intent of finding common features of the virus harbored in them that may have given rise to a potent NAb response. Viral evolution and quasispecies complexity in chronically infected subjects clearly were potential confounding factors; the single sequence used was randomly selected from a complex viral population within each individual and may not reflect the form of the Env that gave rise to the NAbs of interest in the serum samples. Indeed, assuming that the NAb response during chronic infection is driven by multiple viral variants, these confounding factors limit our ability to identify genetic signatures. Despite this, statistically significant signatures were revealed based on an analysis of sequences from a single Env clone from a single time point from each of 69 individuals, indicating a detectable consistency of signal across the population. Notably, despite scanning the full Env, these signatures were focused on a single biologically interesting region, the CoRbs. An unresolved issue that is an inherent consequence of this signature-defining strategy is the uncertainty regarding whether the signature amino acids reflect common features that were useful for stimulating potent NAb responses, or if instead they reflect common patterns of escape from the NAb responses in the potent sera. Experimental comparisons to resolve this are underway; strains that retain the signature positions that are associated with potent sera, like CH0219.e4 and CH080510.e.p2 (Fig. 7), are particularly interesting candidates for immunogenicity testing.

The fact that five of the six signature sites identified, with one false positive expected, were in the CoRbs of gp120 suggests an important role for this region in generating high titers of broadly NAb responses. This region is comprised of elements of the bridging sheet and adjacent surfaces from the outer domain of gp120, including the V3 loop, that undergo conformational changes and become exposed upon CD4 binding as an intermediate step in the membrane fusion process [61], [96], [97], [102]–[104]. It is possible that in some cases CD4i-specific mAbs contribute directly to potent cross-neutralizing ability [87], [95]. The CoRbs is one of the most highly conserved and protected domains on gp120 [86]. Rare variants of HIV-1 exist that exhibit spontaneous exposure of CD4i epitopes; these strains tend to infect cells independently of CD4 and to be highly sensitivity to neutralization by CoR-specific antibodies [105], [106]. Owing to the presence of such antibodies in HIV-1-infected individuals [86], [87], [95], a mechanism of CD4-induced exposure of the CoRbs serves as an effective strategy to evade humoral immunity — a strategy that is aided by steric constraints that prevent anti-CoR antibodies from gaining accessing to their epitopes at the virus-cell interface [107]. In a systematic thermodynamic analysis by Kwong et al., in which 20 antibodies were categorized according to where they bind on the gp120 surface, it was found that 6 of 7 antibodies that bind gp120 at its receptor and coreceptor binding sites exhibited unusually high binding entropy (including 17b that binds to CoRbs) [21]. Therefore, the signature sites identified here in the CoRbs might play an indirect role in neutralization by antibodies that induce large conformational changes in gp120.

The question naturally arises as to why a region of gp120 that is so heavily guarded and difficult to target by NAbs registered in our analysis as a key determinant of potent NAb responses in HIV-1-infected individuals. One possibility is that the CoRbs of gp120 has vulnerabilities that are only beginning to be recognized. For example, using a novel combination of epitope mapping techniques, Li *et al.* [95] reported evidence that CoRbs-specific antibodies contributed to the broadly cross-reactive neutralizing activity of serum from two HIV-1 infected individuals. In addition, CoRbs residues were implicated by alanine scanning mutagenesis as being involved to a minor extent in the epitopes for two newly described broadly neutralizing mAbs [50]. Also, vaccine-elicited CoRbs-specific antibodies correlated with viremia control in a simian-human immunodeficiency virus (SHIV) challenge model in nonhuman primates [108]. It also seems possible that amino acid residues in key positions in the CoRbs of gp120 modulate the conformation of adjacent regions, such as the CD4bs, much the same as conformational changes induced by gp120-CD4 binding modulate the CoRbs. Limited sequence variability in the CD4bs [109], [110] makes this an attractive target for NAb-based vaccines. Indeed, studies have shown that the CD4bs is targeted by broadly NAbs in sera from some HIV-1-infected individuals [51].

It remains to be determined whether the genetic signatures of potent NAb responses identified here contribute to the immunogenicity as well as antigenicity of Env. By design we were attempting to resolve signatures that impacted Env immunogenicity in natural infection. Clearly, strong antigenicity alone is generally not sufficient for the elicitation of NAbs [28]–[30], [36]–[39]. Other requirements may need to be met before B cells can be stimulated to produce NAbs against certain epitopes of interest. Although very little is known about what these requirements might be, proper Env configuration for B cell recognition and antibody affinity maturation should be considered. It will be interesting to test novel Env immunogens that naturally contain the genetic signatures identified in our study, or that introduce these signatures experimentally. At the very least, our findings suggest that greater attention should be paid to the CoRbs of gp120 when designing novel vaccine immunogens.

## Materials and Methods

### Viruses, serum samples and mAb b12

All viruses were used as molecularly cloned Env-pseudotyped viruses that expressed the entire gp160 of the designated strain. The multisubtype panel of viruses used for analysis of b12 neutralization is described in Tables S1 and S2. The 25 viruses used to assess the neutralizing activity of HIV-1-positive serum samples were isolated from sexually acquired infections and were sampled early in infection to closely resembled transmitted/founder viruses. Among these, isolates 6535.3, QH0692.42, SC422661.8, PVO.4, AC10.0.29 and RHPA4259.7 belong to a recommended panel of subtype B reference strains [111]. Isolates Du156.12, Du172.17, Du422.1, ZM197M.PB7 and ZM214M.PL15 belong to a recommended panel of subtype C reference strains [112]. Isolates Q23.17, Q842.d12, Q168.a2, Q259.d2.17, Q461.e2 and Q769.d22 are subtype A reference strains [113]. Isolates BB1006-11.C3.1601, BB1054-07.TC4.1499, 700010040.C9.4520 and WEAU-d15.410.787 are subtype B clones that were confirmed by single genome amplification (SGA) and sequencing analysis to be true transmitted/early founder Envs [56], as were C subtype isolates Ce1086_B2, Ce0393_C3, Ce1176_A3 and Ce2010_F5 [114]. These latter 25 viruses utilized CCR5 as their major coreceptor and were considered to possess a tier 2 neutralization phenotype [115].

Serum samples were obtained from HIV-1-infected subjects who were enrolled in clinical protocols of the Center for HIV/AIDS Vaccine Immunology (CHAVI). All subjects were chronically infected at the time of enrollment. The precise length of time of infection was not known. The mAb b12 was provided by Quality Biologicals, Inc. (Gaithersburg, MD) as a complete IgG molecule.

### SGA amplification and sequencing of gp160 genes

The SGA methods used here were described previously [116] and result in sequences that are not corrupted by recombination during amplification. Viral RNA was prepared from 400 µl of patient plasma and eluted into 60 µl of elution buffer using EZ1Virus Mini Kit V2.0 (Qiagen, Valencia, CA). Viral cDNA was prepared with 20 µl of vRNA and 80 pmol of primer 1.R3.B3R (5′-ACTACTTGAAGCACTCAAGGCAAGCTTTATTG-3′) in a 50 µl volume using Superscript III (Invitrogen; Carlsbad, CA). SGA of the cDNA was performed using nested PCR to obtain the *rev/env* cassette and to avoid artificial recombination and resampling of the viral genomes [117]. The cDNA was diluted 1∶3, 1∶9 and 1∶27 (8 reactions per dilution) to determine a dilution with a positive rate of 20% or less. Each diluted cDNA (1 µl) was used for the first round amplification with primers 07For7 (5′CAAATTAYAAAAATTCAAAATTTTCGGGTTTATTACAG-3′) and 2.R3.B6R (5′-TGAAGCACTCAAGGCAAGCTTTATTGAGGC-3′). First round PCR was carried out with 1 unit of Platinum Taq Polymerase High Fidelity (Invitrogen; Carlsbad, CA) and 10 pmol of each primer in a 20 µl volume. First round PCR products (2 µl) were used for a second round of PCR with primers VIF1 (5′-GGGTTTATTACAGGGACAGCAGAG-3′) and Low2c (5′-TGAGGCTTAAGCAGTGGGTTCC-3′). The second round PCR used 2.5 units of Platinum Taq Polymerase High Fidelity and 20 pmol of each primer in a 50 µl volume. PCR thermocycling conditions were as follows for both rounds of PCR: one cycle at 94°C for 2 minutes; 35 cycles of denaturing step at 94°C for 15 seconds, an annealing step at 60°C for 30 seconds, an extension step at 68°C for 4 minutes, and one cycle at 68°C for 10 minutes. PCR products were visualized on a 1% agarose gel and purified with the QiaQuick PCR Purification kit (Qiagen; Valencia, CA). Sequence analysis of *env* PCR products was performed on both DNA strands by cycle-sequencing and dye terminator methods using an ABI 3730×l genetic analyzer (Applied Biosystems; Foster City, CA). Individual overlapping sequence fragments for each *env* SGA were assembled and edited using the Sequencher program 4.7 (Gene Codes, Ann Arbor, MI). Subtyping analysis was initially performed using SIMPLOT [118]. All sequences were further validated with RIP and HIV Blast (www.hiv.lanl.gov). Subtyping and recombination discrepancies between the methods were carefully considered and resolved. The single SGA Env sequence obtained from each of the HIV-1 positive individuals with potent or weak neutralizing antibody responses was sampled at random. GenBank accession numbers are provided in the supplementary tables.

### Neutralization assay

Neutralization was measured as reductions in luciferase (Luc) reporter gene expression after a single round of infection with Env-pseudotyped viruses as described [111]. Briefly, 200 TCID50 of virus was incubated with serial 3-fold dilutions of test sample in duplicate in a total volume of 150 µl for 1 hr at 37°C in 96-well flat-bottom culture plates. Freshly trypsinized TZM-bl cells (10,000 cells in 100 µl of growth medium containing 37.5 µg/ml DEAE dextran) were added to each well. One set of control wells received cells plus virus (virus control) and another set received cells only (background control). After a 48-hour incubation, 100 µl of cells was transferred to a 96-well black solid plates (Costar) for measurements of luminescence using the Britelite Luminescence Reporter Gene Assay System (PerkinElmer Life Sciences). Neutralization titers are either the 50% inhibitory dilution (ID50, serum samples) or 50% inhibitory concentration (IC50, mAb b12) at which relative luminescence units (RLU) were reduced by 50% compared to virus control wells after subtraction of background RLUs. Assay stocks of molecularly cloned Env-pseudotyped viruses were prepared by cotransfecting 293T/17 cells with an Env-expressing plasmid and an env-minus backbone plasmid (pSG3Δenv) as described [111].

### Definitions of neutralization sensitivity

To conduct Env sequence signature analyses with the goal of identifying mutational patterns that correlate with neutralization phenotypes, we first needed to define neutralization phenotypes. For mAb b12, we initially defined the Envs based on whether or not a 50% reduction in RLU could be achieved at the highest concentration of b12 used; if not, the Env was considered b12 resistant. Some Envs were tested up with to 50 ug/ml of b12, however others were only tested up to 25 ug/ml (Table S2); 9 of the 251 samples had a detectable neutralization response between 25 and 50 ug/ml. Thus did the signature analysis two ways, either treating any result over 25 ug/ml as negative, or treating any positive result as positive; the results were essentially the same either way, and the results presented are based on treating any detected response as positive. This provided a Boolean neutralization sensitive/resistant phenotype to use as a basis for comparing the 251 Envs tested with b12. Later, we compared the levels of neutralization-sensitivity with the patterns in the b12 signature sites by using IC50 values.

Defining a serological phenotype based on a profile of potency of neutralization against a panel of viruses was more complex. We first needed to group HIV-1-positive serum samples that exhibited similar neutralization profiles against a panel of 25 viruses. To achieve this, we used a k-means clustering strategy with two added statistics to assess the robustness of the clusters, factoring in both the uncertainty that results from limited sampling and inter-assay variability (the impact of experimental noise was explored using a smooth bootstrap). Sampling limitations were explored by re-sampling either by rows or columns 1000 times, using a random-with-replacement bootstrap strategy. The impact of inter-assay variation was explored by a smooth bootstrap, re-sampling from a Gaussian model of noise centered on zero and based on a limited number of repeat data values. Noise was adding back to the original scores based on the model. We then re-estimated the k-means clusters 1000 times with noise added back [119]. Using these two strategies we found that no more than k = 3 distinctive clusters of sera were statistically justified, in that 2 or more sera were assigned to each of the three clusters with 90% confidence. Defining more than k = 3 clusters was not justified using this criteria. Sera that were not assigned to a cluster 90% of the time were considered indeterminate; clustering patterns were generally more sensitive to sampling than inter-assay variability. To describe the NAb reactivity pattern of the 3 sera clusters in a Boolean framework (we are limited to two categories, high versus low) for signature pattern analyses, we compared Envs that were members of each of the robust serological clusters to all other Envs in the study. For example, we compared the Env sequences associated with the strongest sera (cluster III) to the remaining Envs by combining those that were in clusters I and II and those that were poorly resolved. In a second analysis, we set k = 2 and compared just the statistically robust high and low clusters, excluding the intermediate values from the comparison.

### Computational methods: alignments, phylogenetic and signature analyses

Alignments used for signature analysis were generated with GeneCutter (www.hiv.lanl.gov), which builds on a HMMER base alignment strategy [120] to provide codon-aligned DNA for phylogenetic and signature analysis. Because hypervariable regions are very difficult to align and compare objectively, we excluded all positions in the alignment that contained more than 10% gaps from the analysis [54]. In practice, this means the difficult to align hypervariable regions and rare insertions were all excluded. Thus, the correlations we find (listed in Tables 1 and 5) are focused in regions of Envelope that are readily aligned, and not a consequence alignment artifacts or impacted by the alignment strategy, with the possible exception of site 186, which borders a hypervariable region in V2.

Phylogenetically corrected methods were used to identify all signature sites; the contingency table method illustrated in Fig. 1 and Fig. 6 was described in detail in [54]. The reason phylogenetic corrections are critical is that observed patterns in data can result either from correlations imposed by the initial historical emergence of a lineage of viruses (founder effects), or in the case of HIV-1, a consequence of recent biological interactions. Not accounting for founder effects can lead to erroneous statistical conclusions [52]. While there are many recombinant sequences included in the tree (as with essentially all HIV population trees), limiting the accuracy of the reconstruction of the evolutionary history, the phylogenetic corrections utilized for signature analysis are, however, dependent only on the local region of the tree and the ancestral states near the tips of the branches, reducing the impact of inter-subtype recombination on the analyses. We implement are ancestral reconstruction through maximum likelihood phylogenies, using code originally based on Gary Olsen's fastDNAML [121], with a GTR model with a likelihood estimate of rate variation per site, and adapted to given ancestral states at all nodes [54].

While a maximum likelihood tree framework enables us to model the ancestral state of the virus immediately preceding the tip, neutralization sensitivity data exists only at the terminal tips of the tree, and we do not attempt to infer phenotypic information at internal nodes. To look for amino acid correlations with phenotype, we tested each position in Env that contained fewer than 10% gaps, for associations of the phenotype of interest based on each amino acid considered alone, any combination of two amino acids at each site, and all combinations of two amino acids at two adjacent sites. We also tested for the preservation or loss of glycosylation sequon motifs at particular positions in the alignment. Finally, we included all possible sets of amino acids in up to 3 positions within four targeted and defined regions of interest (the structurally defined b12 binding site, the CD4bs, the CoRbs, and the MPER regions, summarized in supplement Table S3). More extensive tests of combinations of sites throughout Env would not have been useful because of power issues given multiple testing. Precisely the same algorithm and series of tests were applied to both the b12 sensitivity data and the data relating to potent neutralization.

A large sample size is essential to power explorations of associations between phenotype and mutational patterns. Thus phylogenetic reconstruction can be challenging because the number of possible relationships grows factorially with the number of sequences sampled. To improve our maximum likelihood tree reconstructions, we adapted our phylogenic code [52] to a newly high performance computing platforms (http://www.lanl.gov/roadrunner/). Trees were run using 25 global rearrangements for up to three days on 512 Cell-accelerated processors, until the likelihood scores no longer improved. The final signature results are relatively robust to the tree however, and a rapidly obtained PHYML tree yielded sound signature results, as discussed in the Results. Access to parallel computing resources at Los Alamos National Laboratory also facilitated protein modeling of loop structures and other computationally intensive and repetitive tasks, such as combinatorial signature analysis and q value calculations.

Felsenstein first developed the method of phylogenetically independent contrasts many years ago [122] to address similar problems, i.e., obtaining phylogenetic corrections when looking for correlations of mutational patterns with quantitative data. We were indeed interested in exploring associations between several quantitative measures and neutralization phenotypes, in particular both loop lengths and the total number of PNLGs in hypervariable regions. However, because hypervariable loop lengths and the number of PNLGs vary rapidly within infected individuals, and so are changing on a time scale much faster then the time scale reflected in the population-based trees, a phylogenetic correction at the population level was deemed not essential in this framework. Thus, for testing the impact of loop lengths and numbers of glycosylation sites, simple Spearman correlation tests were performed.

### Conditional mutual information (CMI) based signatures

Conditional mutual information (CMI) was used as a second computational method to identify positions that exhibit an association between mutation and phenotype (neutralization sensitivity) that is independent of phylogenetic lineage. CMI [123] generalizes the conventional mutual information measure [123] that quantifies the association between two objects, e.g., mutation and phenotype. CMI also quantifies the association between two objects but it conditions the association on a third object, in this case the ancestral state. CMI sums over the associations conditioned on different ancestral state amino acids, and so is potentially more sensitive for detecting associations than the contingency table analysis that involves one ancestor state at a time. On the other hand, if the biological signal exists only for some ancestral states and not others, the extra noise added may reduce the power of the test. The statistical significance of a CMI value at any given position was assessed by fixing the ancestral state to each candidate ancestor state in turn, and permuting the relation between mutation and phenotype 1000 times in order to break any potential association. The distribution of CMI values for such permuted data was used to determine p-values, and q-values were obtained from these using the method of Storey and Tibshirani [124]. As with the Fischer's exact test signatures, a cutoff of q<0.2 was used to identify statistically interesting sites, such that a 20% false discovery rate was expected among the identified signatures.

### Ensemble learning technique using classification trees

To model sequence changes across sites, an ensemble learning technique using classification trees was employed [125]. As with the CMI and contingency table approaches, a sensitive/resistant neutralization category was compared to phylogenetic signals. This neutralization quantity indicates when a virus is neutralized by a fixed amount of b12 antibody. A change observed between an observed amino acid and the corresponding position in the inferred parent sequence provides one phylogenetic signal. Changes toward or away from each observed or inferred amino acid across all of the envelope protein sequences served as the set of phylogenetic signals. Signals are conditioned on the ancestor amino acid; thus, any given position can be an instance of the signal, not an instance of the signal, or not applicable for the signal.

To form a decision tree, a signal was first identified that best separated sequences into resistant and sensitive neutralization sets. Each set was then partitioned into two more sets using further signals that best track the neutralization phenotype. This refinement procedure was repeated until no additional signals improved the classification. It was necessary that the classification tree handle the absence of signals as well as their Boolean state in order to avoid phylogenetic artifacts. Prediction was performed by taking a tree and following a main signal, secondary signal, tertiary signal, and so on, according to signal values derived from new data. Even in the absence of mutational signals, a decision tree would still provide a prediction on the basis of whether resistant or sensitive viruses were more common in a training set.

It is conceivable that coordinated mutations or reversions could occur in a universal way across viruses (case 1). Alternatively, the interplay of viruses and hosts could result in different patterns of coordinated sequence change (case 2). To address the possibility that there can be context dependence on unmeasured quantities (i.e., virus behavior groups formed by some unknown process), we randomly sampled a subset of the full training data (75%) when building decision trees, performing 140 interactions of decision tree building with different training set samples. 75% of the data was chosen as a trade-off between statistical power (ability to see any group behavior) and diversity (ability to see several groups). We chose 140 iterations for computational feasibility. Evaluation of the performance of the decision tree models needed to be separate from the construction of the training data. Thus, before iterating the training set sampling and tree building, we reserved 5 sensitive and 5 resistant viruses for testing purposes. Good models from the 140 decision tree builds were defined as those models that perform better than 60% (instead of the expectation of 50% for random guesses) on this reserve dataset.

Any one of the 140 training samples and resulting decision trees could represent either case 1 or case 2, as described above. Therefore, the full process of reserving a random test set and generating 140 models to ‘hit’ each test set was iterated 32 times. For each test set, we obtained on average 10 of the 140 models predictive to at least 60% accuracy. A majority vote of these model predictions was noted for each test set. A “majority vote” was conducted across the 32 test sets to provide the final neutralization prediction. Finally, we identified mutational patterns that recurred most often at the top-level splits in the subset of good models across all runs. These patterns provided another strategy for defining amino acid signatures that correlate with neutralization phenotype (Table 1), and these were a subset of the sites defined by the most common highest level splits; this method only defined a subset of the statistically promising sites defined by the basic Fischer's exact methods.

Unlike other decision forest or bootstrap aggregation approaches (a.k.a. bagging) [126], we cross-validated within the training set and pruned back the trees before using them. This may limit overall accuracy, but it has the advantage that any decision tree model could be interpreted without overtly over-fitting a particular training data set.

### Structural mapping of signature positions

For structural mapping in gp120, three different structures were used. We used a structure with loops modeled when residue positions in loops needed to be shown. In this structure, the core of gp120 corresponded to the X-ray structure of CD4-bound YU2 gp120 [127], with variable loops V1, V2 and V3 modeled for visualization purpose as described previously [128]. For signature positions in the b12 binding surface of gp120, we used the X-ray structure corresponding to the PDB code 2NY7 [35]. Finally, for spatial mapping of the signature positions in the CD4i region, we used the X-ray structure with a PDB code, 1RZK, [127] that was solved with a CD4-17b complex. In one instance a three-dimensional structure of gp41 was used to suggest the possibility of allosteric effects within the gp120-gp41 complex. This latter gp41 structure was homology-modeled based on the NMR structure of SIV-1 gp41 structure [129]. Signature positions were mapped onto this structure based on the alignment of sequences with respect to HXB2. The positional numbering refers to HXB2. Three-dimensional images were generated using VMD [130].

### Validation of b12 signatures

A holdout set of 56 pseudotyped Envs, for which the b12 sensitivity was known but withheld from the analysis team, was kept aside as a fully blinded test set to determine if we could predict the b12 phenotype of Env-pseudotyped viruses based on either just signature amino acid positions or the ensemble learning strategy across full Env. The training and test set of Envs are included in the phylogenetic tree shown in Fig. 1; viruses known to be b12-sensitive are magenta, those known to be b12-resistant are dark grey, and those used as a blinded test set are light gray. Several strategies to predict phenotype were employed, including the simple requirement of at least 4 sensitive and no more than 1 resistant amino acid in the 7 signature sites, a logistic regression based on the 7 signature sites, and the ensemble learning strategy based on the full Env alignment. A prediction of b12 sensitivity or resistance was made based on all three strategies (Tables 3 and 4, Table S2) for each of the 251 original training sequences and 56 test sequences.

### Ethics statement

The research presented here was approved by the Duke Institutional Review Board, and the human data was analyzed anonymously using de-identified preexisting samples.

## Supporting Information

Figure S1 An alignment of the three additional sites that were identified by the CMI method.
(0.25 MB TIF)

Figure S2 The correlation between signature sensitivity score and b12 sensitivity.
(0.11 MB TIF)

Table S1 This table includes a description of the 251 sequences of the pseudotyped viruses used for defining the b12 signatures, as well as the 56 sequences included in the blinded set used to test whether the signature could be used to predict b12 sensitivity. It includes strain name, HIV-1 subtype or recombinant status, country of origin, year, Fiebig stage, modes of transmission, specimen and GenBank accession number. A blank means the field is unknown.
(0.07 MB XSLX)

Table S2 This table includes a description of the 251 sequences of the pseudotyped viruses used for defining the b12 signatures, as well as the 56 sequences included in the blinded set used to test whether the signature could be used to predict b12 sensitivity. It contains b12 neutralization potency, b12 IC50 value, the prediction of b12 status based on the ensemble learning technique and the logistic regression, signature site amino acids in each sequence and the total number of signatures that are susceptible and resistance in each sequence that was utilized in the prediction using simple cumulative pattern. A blank means the field is unknown.
(0.41 MB XLS)

Table S3 Sets of sites used for deeper combinatorial analyses of signatures.
(0.03 MB DOC)

Table S4 Summary of charged residues in the gp120 core structure. Qualitative evaluation of all acidic residues in the recent X-ray structure of b12-bound to the JRFL gp120 [35] that was used in the electrostatic potential calculations.
(0.04 MB DOC)

Table S5 A list of all sites that co-vary with b12 signature sites. All sites are found to co-vary in a contingency table analysis with a q-value<0.2. Co-variation sets among signature sites are highlighted in bold or underlined.
(0.04 MB DOC)

Table S6 HIV-1-positive serum samples used for signature analysis. Single SGA Env clones were sequenced from each sample. All samples were taken during chronic infection, at the same time the sample was tested for cross-reactive neutralizing antibodies. All sequences have been submitted to GenBank (in progress).
(0.14 MB DOC)

Table S7 HIV-1 strains used for NAb assays to identify signatures in serum-derived Env sequences.
(0.06 MB DOC)
