# Supplementary figures and images for: Genetic Signatures in the Envelope Glycoproteins of HIV-1 that Associate with Broadly Neutralizing Antibodies
Source: PLoS Comput Biol. 2010 Oct 7;6(10):e1000955. doi: 10.1371/journal.pcbi.1000955 (PMC2951345; doi:10.1371/journal.pcbi.1000955)

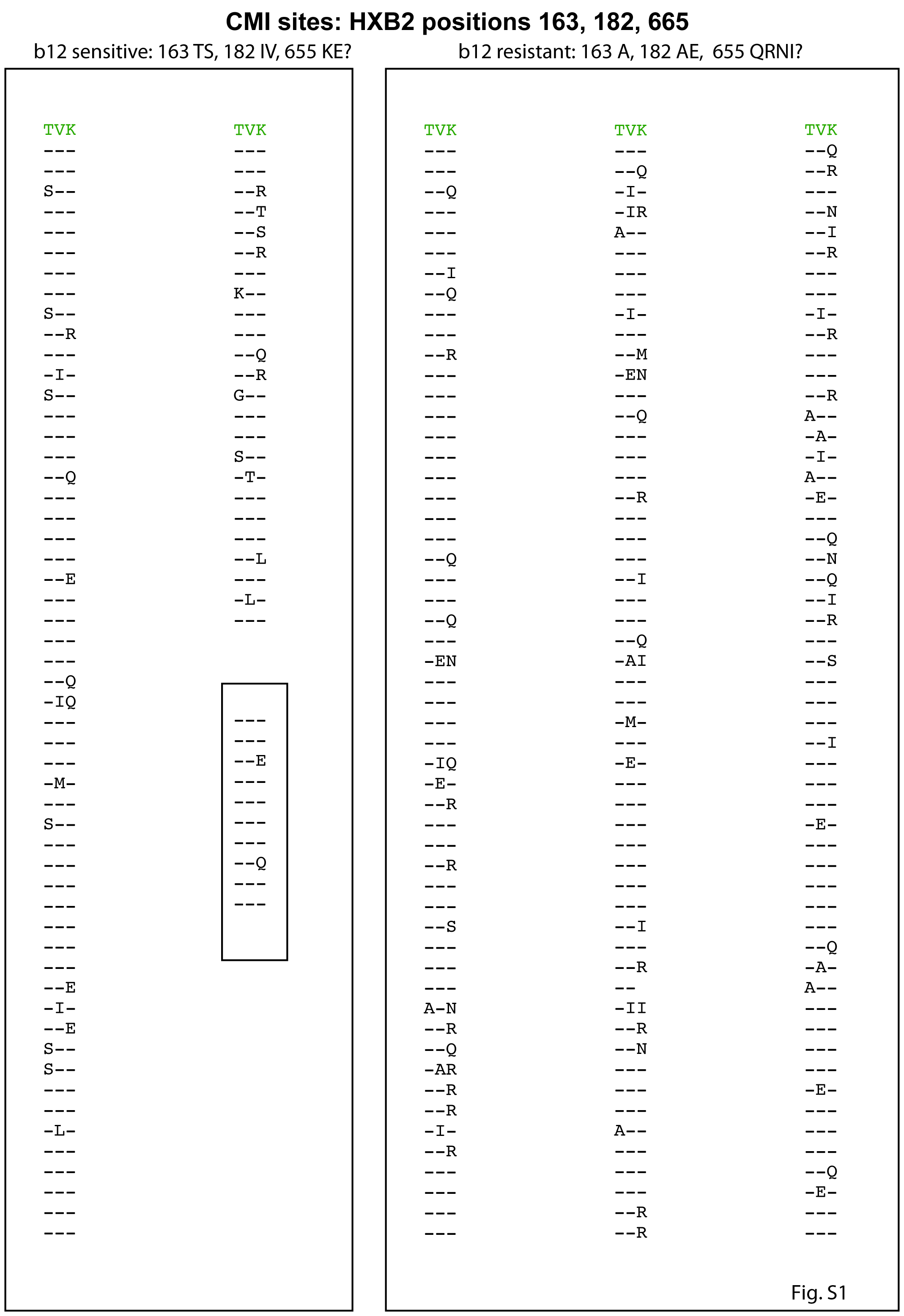

Supplement: Figure S1 — An alignment of the three additional sites that were identified by the CMI method. (0.25 MB TIF) [file pcbi.1000955.s001.tif]

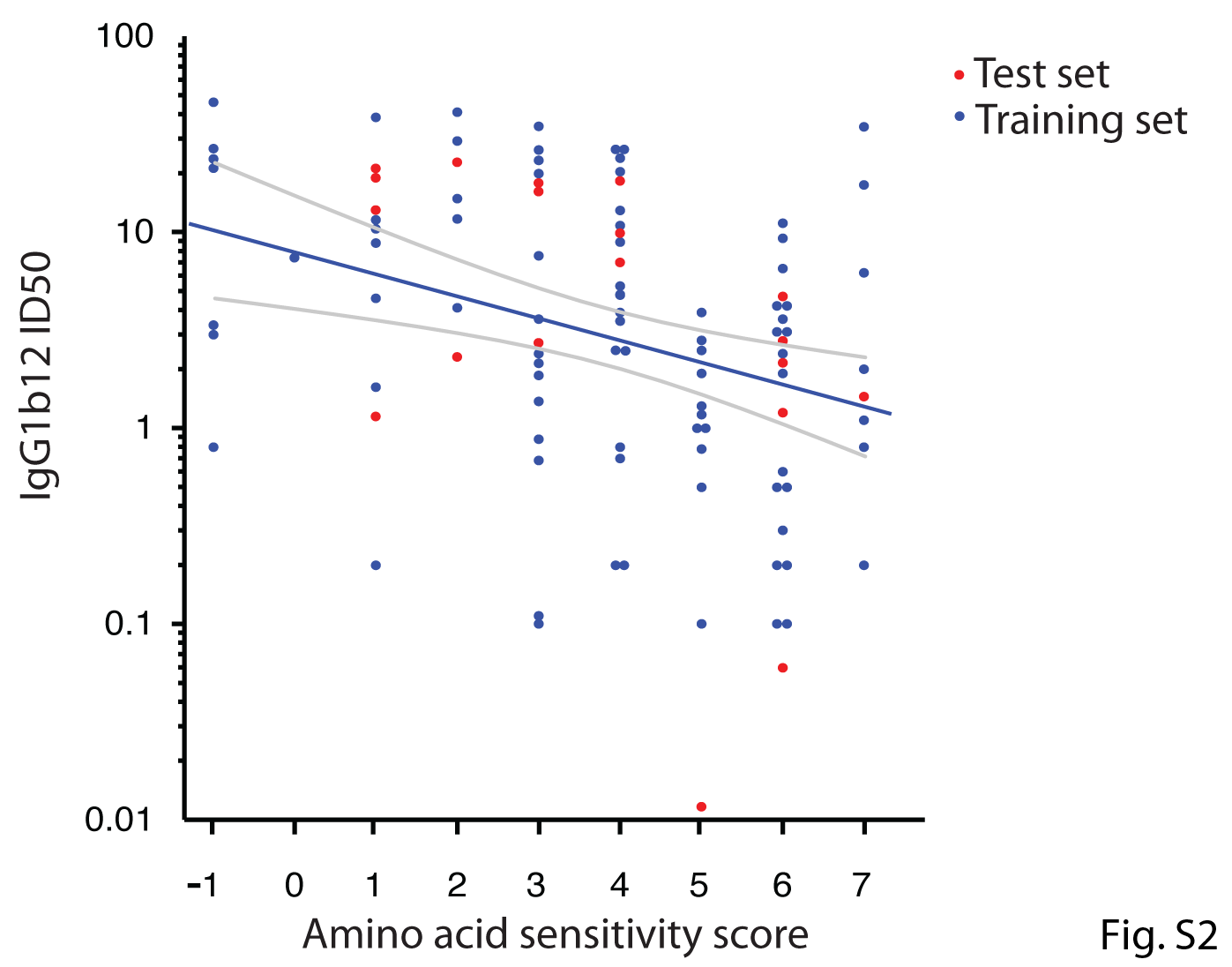

Supplement: Figure S2 — The correlation between signature sensitivity score and b12 sensitivity. (0.11 MB TIF) [file pcbi.1000955.s002.tif]
