# Supplementary material for: Genetic Signatures in the Envelope Glycoproteins of HIV-1 that Associate with Broadly Neutralizing Antibodies
Source: PLoS Comput Biol. 2010 Oct 7;6(10):e1000955. doi: 10.1371/journal.pcbi.1000955 (PMC2951345; doi:10.1371/journal.pcbi.1000955)
Supplement: Table S3 — Sets of sites used for deeper combinatorial analyses of signatures. (0.03 MB DOC) [file pcbi.1000955.s005.doc]

**Table S3. Sets of sites used for deeper combinatorial analyses of signatures.**

| **Region^1^** | **Env glycoprotein** | **Amino acid positions (HXB2 numbering)** | **Reference(s)** |
| --- | --- | --- | --- |
| b12 epitope | gp120 | A281, S364, S365, G367, P369, T373, Y384, N386, P417, C418, R419, D474 | [35] |
| CD4bs | gp120 | E102, H105, T123, P124, L125, C126, V127, S128, L129, T194, S195, C196, N197, T198, V255, S256, T257, V275, T278, D279, N280, A281, K282, T283, S364, S365, G366, G367, D368, P369, E370, I371, V372, S375, Y384, I424, N425, M426, W427, Q428, K429, V430, G431, K432, L453 | [60] |
| CoRbs | gp120 | K117, K121, K207, N377, E381, R419, I420, K421, Q422, P438, R440, G441, R444 | [95, 96] |
| MPER | gp41 | Contiguous positions 664-680: D664, K665, W666, A667, S668, L669, W670, N671, W672, F673, D674, I675, T676, N677, W678, L679, W680 | [87, 131] |

^1^b12 epitope, the region of gp120 that is bound by mAb b12; CD4bs, the CD4 binding site; CoRbs, the CCR5 coreceptor binding site; MPER, membrane proximal external region. The entire protein was scanned for simple signatures, but for more complex signatures (multiple amino acids per position and multiple positions in combination) to make the analyses computationally feasible, only regions of known biological relevance were scanned. We also looked at preservation or loss of glycosylation sequons (potential N-linked glycosylation sites PNLGs) in conjunction with neutralization susceptibility, testing for the acquisition or loss of the amino acid pattern Nx[ST], where N is an Asp, x is any amino acid, and [ST] is either a Ser or Thr.
