# Supplementary material for: Genetic Signatures in the Envelope Glycoproteins of HIV-1 that Associate with Broadly Neutralizing Antibodies
Source: PLoS Comput Biol. 2010 Oct 7;6(10):e1000955. doi: 10.1371/journal.pcbi.1000955 (PMC2951345; doi:10.1371/journal.pcbi.1000955)
Supplement: Table S4 — Summary of charged residues in the gp120 core structure. Qualitative evaluation of all acidic residues in the recent X-ray structure of b12-bound to the JRFL gp120 [35] that was used in the electrostatic potential calculations. (0.04 MB DOC) [file pcbi.1000955.s006.doc]

**Table S4. Summary of charged residues in the gp120 core structure.** Qualitative evaluation of all acidic residues in the recent X-ray structure of b12-bound to the JRFL gp120 [35] that was used in the electrostatic potential calculations.

| **Residue** | **Salt bridge partner** | **Conservation** | **Correlated Mutations** |
| --- | --- | --- | --- |
| E83 | None | Conserved | - |
| E91 | K284 | Conserved | - |
| D99 | R480 | - | - |
| E102 | R480 | D/E/Q/N | - |
| E106 | - | A/T/Q/E/K | - |
| D107 | - | Conserved | - |
| D113 | - | Conserved | - |
| E211 | - | D/Q/T | - |
| E267 | K231 | - | 230/231 |
| E268 | - | - | - |
| E269 | K348 | - | 348 |
| D279 | K282 | D/N/S/K | - |
| E293 | K337 | - | 337 |
| E351 | K348 | - | 348 |
| D368 | - | Conserved | - |
| E370 | - | Conserved | - |
| E381 | - | Conserved | - |
| D412 | R335 | - | 335 |
| D457 | b12 interface | Conserved | - |
| E464 | K357 | - | - |
| E466 | R456 | - | 456 |
| D474 | R480 | No charge flip | - |
| D477 | R480 | Conserved | - |
| E482 | K485 | Variable | - |
| E492 | K490 | - | K490 |
