# Supplementary material for: Genetic Signatures in the Envelope Glycoproteins of HIV-1 that Associate with Broadly Neutralizing Antibodies
Source: PLoS Comput Biol. 2010 Oct 7;6(10):e1000955. doi: 10.1371/journal.pcbi.1000955 (PMC2951345; doi:10.1371/journal.pcbi.1000955)
Supplement: Table S5 — A list of all sites that co-vary with b12 signature sites. All sites are found to co-vary in a contingency table analysis with a q-value<0.2. Co-variation sets among signature sites are highlighted in bold or underlined. (0.04 MB DOC) [file pcbi.1000955.s007.doc]

**Table S5: A list of all sites that co-vary with b12 signature sites.** All sites are found to co-vary in a contingency table analysis with a q-value < 0.2. Co-variation sets among signature sites are highlighted in bold or underlined.

| **b12 signature site^1^** | **Env glycoprotein** | **Co-varying site(s) ^1^** |
| --- | --- | --- |
| 163 | gp120 | None |
| 173 | gp120 | 8, 10, 47, 150, 151, 155, 156, 172, 181, 292, 305, 340, 364**,** 707, 732, 813 |
| 182 | gp120 | None |
| **185** | gp120 | 20, 29, 97, 130, 134, 152, 200, **268,** 271, 275, 300, 304, 311, 320, 340, 397, 429, 476, 490, 496, 588, 595, 607, 633, **655,** 724, 792, 821, 837 |
| **268** | gp120 | 4, 5, 108, 121, 149, 159, 169, 170, 178, 183, 184, **185,** 189, 202, 231, 271, 273, 297, 341, 348, 412, 429, 430, 462, 464, 502, 629, 641, 727, 777, 840, 855 |
| 364 | gp120 | 173 |
| 369 | gp120 | None |
| 461 | gp120 | 133, 336, 464, 515, 779, 831 |
| 651 | gp41 | 80, 84, 169, 429^2^, 432, 602, 798, 817, 822 |
| **655** | gp41 | **185** |

^1^All site positions are based on HXB2 numbering.
