# Supplementary material for: Genetic Signatures in the Envelope Glycoproteins of HIV-1 that Associate with Broadly Neutralizing Antibodies
Source: PLoS Comput Biol. 2010 Oct 7;6(10):e1000955. doi: 10.1371/journal.pcbi.1000955 (PMC2951345; doi:10.1371/journal.pcbi.1000955)
Supplement: Table S6 — HIV-1-positive serum samples used for signature analysis. Single SGA Env clones were sequenced from each sample. All samples were taken during chronic infection, at the same time the sample was tested for cross-reactive neutralizing antibodies. All sequences have been submitted to GenBank (in progress). (0.14 MB DOC) [file pcbi.1000955.s008.doc]

**Table S6. HIV-1-positive serum samples used for signature analysis.** Single SGA Env clones were sequenced from each sample. All samples were taken during chronic infection, at the same time the sample was tested for cross-reactive neutralizing antibodies. All sequences have been submitted to GenBank (in progress).

| **Patient code** | **Subtype** | **Sample Country** | **Year** | **sequence name** | **GenBank Accession Number** |
| --- | --- | --- | --- | --- | --- |
| 700010025 | B | USA | 2006 | CH010025.w48.p1 | submitted |
| 704010028 | C | South Africa | 2007 | CH010028.w24.p1 | submitted |
| 700010032 | B | USA | 2006 | CH010032.48.p1 | submitted |
| 703010073 | C | Malawi | 2007 | CH010073.w16.p1 | submitted |
| 703010085 | C | Malawi | 2007 | CH010085.w4.p1 | submitted |
| 706010090 | C | South Africa | 2007 | CH010090.w8.p1 | submitted |
| 700010094 | B | USA | 2006 | CH010094.w48.p1 | submitted |
| 703010098 | C | Malawi | 2007 | CH010098.w16.p1 | submitted |
| 703010102 | C | Malawi | 2007 | CH010102.e.p2 | submitted |
| 700010111 | B | USA | 2006 | CH010111.w48.p1 | submitted |
| 704010124 | C | South Africa | 2007 | CH010124.w24.p1 | submitted |
| 702010141 | C | Malawi | 2007 | CH010141.w12.p1 | submitted |
| 703010167 | C | Malawi | 2007 | CH010167.w8.p2 | submitted |
| 703010180 | C/F | Malawi | 2007 | CH010180.w12.p1 | submitted |
| 704010207 | C | South Africa | 2007 | CH010207.w4.p1 | submitted |
| 704010210 | C | South Africa | 2007 | CH010210.w2.p2 | submitted |
| 701010211 | B | USA | 2008 | CH010211.w2.p1 | submitted |
| 702010259 | C | Malawi | 2008 | CH010259.w16.p1 | submitted |
| 704010273 | C | South Africa | 2007 | CH010273.w4.p1 | submitted |
| 702010293 | C | Malawi | 2008 | CH010293.w8.p1 | submitted |
| 704010298 | C | South Africa | 2007 | CH010298.w12.p1 | submitted |
| 704010301 | C | South Africa | 2007 | CH010301.w12.p1 | submitted |
| 704010316 | C | South Africa | 2007 | CH010316.w16.p1 | submitted |
| 704010327 | C/G | South Africa | 2007 | CH010327.w12.p1 | submitted |
| 704010330 | C | South Africa | 2007 | CH010330.w16.p1 | submitted |
| 704010343 | C | South Africa | 2007 | CH010343.w12.p1 | submitted |
| 704010355 | C | South Africa | 2007 | CH010355.w2.p1 | submitted |
| 704010368 | C | South Africa | 2007 | CH010368.w8.p2 | submitted |
| 706010383 | C | South Africa | 2007 | CH010383.w12.p1 | submitted |
| 704010384 | C | South Africa | 2007 | CH010384.w16.p2 | submitted |
| 704010392 | C | South Africa | 2007 | CH010392.w4.p2 | submitted |
| 704010408 | C | South Africa | 2007 | CH010408.w12.p1 | submitted |
| 704010420 | C | South Africa | 2007 | CH010420.w16.p1 | submitted |
| 702010432 | C | Malawi | 2008 | CH010432.w4.p1 | submitted |
| 702010440 | C | Malawi | 2008 | CH010440.w4.p1 | submitted |
| 704010453 | C | South Africa | 2007 | CH010453.w12.p3 | submitted |
| 704010461 | C | South Africa | 2007 | CH010461.w12.p1 | submitted |
| 704010540 | C | South Africa | 2008 | CH010540.e.p1 | submitted |
| 704010581 | C | South Africa | 2008 | CH010581.e.p1 | submitted |
| 704010605 | C | South Africa | 2008 | CH010605.w12.p1 | submitted |
| 707010175 | A | Tanzania | 2008 | CH0175.e2 | submitted |
| 707010219 | A1 | Tanzania | 2008 | CH0219.e4 | submitted |
| 703010269 | C | Malawi | 2007 | CH0269.e3 | submitted |
| 707010457 | C | Tanzania | 2008 | CH0457.e1 | submitted |
| 705010534 | C | South Africa | 2008 | CH0534.e1 | submitted |
| 707010536 | A1/C | Tanzania | 2008 | CH0536.e2 | submitted |
| 713080024 | B | England | 2008 | CH080024.e.p1 | submitted |
| 713080038 | B | England | 2008 | CH080038.e.p2 | submitted |
| 713080046 | B | England | 2008 | CH080046.e.p1 | submitted |
| 713080052 | B | England | 2008 | CH080052.e.p1 | submitted |
| 713080060 | B | England | 2008 | CH080060.e.p1 | submitted |
| 713080071 | B | England | 2008 | CH080071.e.p2 | submitted |
| 713080087 | B | England | 2008 | CH080087.e.p1 | submitted |
| 713080095 | B | England | 2008 | CH080095.e.p1 | submitted |
| 713080100 | CRF01_AE | England | 2008 | CH080100.e.p1 | submitted |
| 713080117 | A1 | England | 2008 | CH080117.e.p1 | submitted |
| 713080128 | B | England | 2008 | CH080128.e.p1 | submitted |
| 713080134 | B | England | 2008 | CH080134.e.p1 | submitted |
| 713080142 | B | England | 2008 | CH080142.e.p1 | submitted |
| 713080156 | B | England | 2008 | CH080156.e.p1 | submitted |
| 713080169 | B | England | 2008 | CH080169.e.p1 | submitted |
| 713080175 | B | England | 2008 | CH080175.e.p2 | submitted |
| 713080183 | B | England | 2008 | CH080183.e.p1 | submitted |
| 713080191 | B | England | 2008 | CH080191.e.p1 | submitted |
| 713080203 | B | England | 2008 | CH080203.e.p1 | submitted |
| 713080219 | B | England | 2008 | CH080219.e.p2 | submitted |
| 713080225 | B | England | 2008 | CH080225.e.p2 | submitted |
| 713080258 | B | England | 2008 | CH080258.e.p2 | submitted |
| 713080510 | A1 | England | 2008 | CH080510.e.p2 | submitted |
