# Supplementary material for: Genetic Signatures in the Envelope Glycoproteins of HIV-1 that Associate with Broadly Neutralizing Antibodies
Source: PLoS Comput Biol. 2010 Oct 7;6(10):e1000955. doi: 10.1371/journal.pcbi.1000955 (PMC2951345; doi:10.1371/journal.pcbi.1000955)
Supplement: Table S7 — HIV-1 strains used for NAb assays to identify signatures in serum-derived Env sequences. (0.06 MB DOC) [file pcbi.1000955.s009.doc]

**Table S7**. HIV-1 strains used for NAb assays to identify signatures in serum-derived Env sequences.

| **Virus name** | **Subtype** | **Country** | **Year** | **Fiebig stage** | **Mode of transmission** | **Accession** |
| --- | --- | --- | --- | --- | --- | --- |
| 6535.3 | B | USA | 1995 | V | M-M | AY835438 |
| QH0692.42 | B | Trinidad | 1994 | V | F-M | AY835439 |
| SC422661.8 | B | Trinidad | 1994 | IV | F-M | AY835441 |
| PVO.4 | B | Italy | 1996 | III | M-M | AY835444 |
| AC10.0.29 | B | USA | 1998 | III | M-M | AY835446 |
| RHPA4259.7 | B | USA | 2000 | ≤V | M-F | AY835447 |
| BB1006-11.C3.1601 | B | USA | 1997 | III | Sexual | EU289183 |
| BB1054-07.TC4.1499 | B | USA | 1997 | II | Sexual | EU289185 |
| 700010040.C9.4520 | B | USA | 2006 | V | MSM | EU289193 |
| WEAU-d15.410.787 | B | USA | 1990 | II | MSM | EU289202 |
| Du156.12 | C | S. Africa | 1998 | ≤IV | M-F | DQ411852 |
| Du172.17 | C | S. Africa | 1998 | VI | M-F | DQ411853 |
| Du422.1 | C | S. Africa | 1998 | V | M-F | DQ411854 |
| ZM197M.PB7 | C | Zambia | 2002 | ≤VI | F-M | DQ388515 |
| ZM214M.PL15 | C | Zambia | 2003 | ≤VI | F-M | DQ388516 |
| Ce1086_B2 | C | Malawi | 2004 | I/II | Sexual | FJ444395 |
| Ce0393_C3 | C | Malawi | 2003 | IV | Sexual | FJ444215 |
| Ce1176_A3 | C | Malawi | 2004 | I/II | Sexual | FJ444437 |
| Ce2010_F5 | C | Malawi | 2005 | IV | Sexual | FJ444561 |
| Q23.17 | A | Kenya | 1994 | VI | M-F | AF004885 |
| Q168.a2 | AD | Kenya | 1995 | Acute/early | M-F | AF407148 |
| Q259.d2.17 | A | Kenya | 1994 | Acute/early | M-F | AF407152 |
| Q461.e2 | AD | Kenya | 1995 | Acute/early | M-F | AF407156 |
| Q769.d22 | A | Kenya | 1996 | Acute/early | M-F | AF407158 |
| Q842.d12 | A | Kenya | 1994 | Acute/early | M-F | AF407160 |
